# Role of Cardoon (*Cynara* spp.) and Raw Milk Microbiota in Iberian PDO and PGI Small Ruminants’ Milk Cheeses

**DOI:** 10.3390/foods15132359

**Published:** 2026-07-02

**Authors:** Carlos Dias Pereira, Lara Campos, Adélcia Veiga, Susana Pereira-Dias, Marta Henriques

**Affiliations:** 1 School of Agriculture, Polytechnic University of Coimbra, 3045-601 Coimbra, Portugal; sudias@esac.pt (S.P.-D.); mhenriques@esac.pt (M.H.); 2 Research Centre for Natural Resources Environment and Society (CERNAS), Polytechnic University of Coimbra, 3045-601 Coimbra, Portugal; lara.campos@esac.pt; 3 Biomass Conversion and Bioprocess Technology Group, Department of Biotechnology and Biomedicine, Technical University of Denmark, 2800 Kongens Lyngby, Denmark; adelcia.veiga@esac.pt

**Keywords:** Iberian cheeses, small ruminants raw milk microbiota, *Cynara* spp., cardoon, PDO and PGI certification, proteolysis and lipolysis

## Abstract

The Protected Denomination of Origin (PDO) and Protected Geographic Indication (PGI) labels were established to legally protect traditional cheeses, particularly those derived from small ruminants’ milk, through the definition of strict production standards. Nevertheless, the impact of certification has often fallen short of initial expectations in terms of sector valorisation and rural development. Increasing the economic sustainability of traditional small ruminants’ raw milk cheeses requires scaling without compromising their distinctive identity. In this context, increasingly stringent regulations on the hygiene and disinfection practices associated with milk refrigeration have significantly affected the characteristic properties of these cheeses, which are largely shaped by traditional manufacturing practices and the indigenous milk microbiota. This review synthesises distinctive attributes of Spanish and Portuguese PDO/PGI cheeses and emphasises the roles of cardoon (*Cynara* spp.) extracts and small ruminants’ raw milk microbiota in influencing the proteolysis, lipolysis, texture and flavour of such cheeses. Variability in cardoon ecotypes, enzyme activity, and microbial composition strongly affects cheese texture, aroma, and safety. Key challenges include inconsistent coagulant quality, the hygienic constraints associated with raw milk, regulatory limitations, and restricted market access. This review outlines strategies to address these challenges, including the standardisation and selection of elite cardoon ecotypes, improved milk hygiene practices, the development of tailored starter and non-starter cultures, and risk-based regulatory approaches. These measures are crucial to preserve authenticity while ensuring safety and economic resilience, thereby reinforcing the role of Iberian PDO/PGI cheeses in sustaining small ruminant dairy systems and rural economies.

## 1. Introduction

### 1.1. Iberian PDO and PGI Small Ruminants’ Milk Cheeses

Dairy sheep are primarily concentrated in the Mediterranean and Black Sea regions, where cheese is a central component of the diet. By contrast, dairy goats predominate in low-income countries of the Indian subcontinent but are also widely distributed in Southern Europe. Sheep’s and goat’s milk cheeses represent a relevant niche within the European dairy sector. Sheep and goats play a key role in traditional European production systems and rural economies, where their milk is transformed on farm or delivered to dairies to produce traditional cheeses and other regional products with high cultural and economic value [1]. Several European countries have adopted Protected Designation of Origin or Protected Geographical Indication (PDO/PGI) labels to safeguard these products through defined production standards. However, in Portugal, the impact of such certification has not met initial expectations regarding sector valorisation or rural development.

Global milk production has increased steadily, reaching approximately 985 million tons in 2024, with cow’s milk representing the largest share [2]. By contrast, the contribution of small ruminants (i.e., goats and sheep) remains quantitatively limited, with goat’s milk representing approximately 2.0% and sheep’s milk 1.1% of global production [3]. In the European Union (EU), the total milk production was estimated to be 161.8 million tons in 2024, continuing the upward trend observed over the past decade [4]. Most of this milk is processed into manufactured products, namely cheese, with EU production reaching 10.8 million tons in 2024 [5].

Although cheeses produced from mixed milk (e.g., ewe–goat or cow–ewe–goat) are also common, according to Eurostat (codes D7122 and D7123), the EU production of (pure) sheep’s milk cheese accounted for 230 thousand tons in 2024, while goat’s milk cheese represented around 168 thousand tons [5]. These cheeses play an important role in the regional agri-food systems. Sheep’s milk cheese production in Europe is concentrated in a limited number of countries. Spain is the leading producer, with approximately 76.8 thousand tons in 2024, followed by Italy and France, with 72.4 and 67.5 thousand tons, respectively [6]. These countries maintain strong traditions of small-scale cheese production integrated into regional agri-food chains. In Portugal, although milk production volumes are lower, sheep and goat farming remains relevant for family-based systems, particularly in regions such as Beira Interior, where traditional sheep and goat cheeses represent niche products with significant cultural and economic value. These data illustrate the dual dimension of small ruminants in the EU: a limited quantitative contribution to total milk production but a significant qualitative role in high-value traditional products, reinforced by PDO/PGI certification and with strong links to historical agro-pastoral practices.

However, over the past 25 years, sheep and goat farming in Portugal has declined sharply. Between 1999 and 2009, the number of farms decreased by 40%, while the number of livestock fell by 22% [7]. In parallel, the number of dairy companies engaged in certified cheese production has also declined [8]. Reversing this trend requires new strategies to mitigate flock reduction, attract new producers, and revitalise rural areas facing depopulation. In this context, a comprehensive evaluation of Iberian PDO and PGI cheeses produced from small ruminant milk, aligned with the recent findings about their distinctive characteristics and current challenges, may support the development of policies aimed at strengthening these systems and enhancing rural sustainability.

Freitas et al. [9] conducted a review on the technological and sensory characteristics of Iberian PDO cheeses produced from ewe and/or goat milk. However, substantial advances have been made over the past 25 years. The present review critically examines how enzymatic and microbial dynamics influence proteolysis, lipolysis, texture, and flavour, while highlighting variability in coagulant activity and microbiota as key sources of inconsistency and safety concerns. The physicochemical and microbial characteristics of these cheeses are also highlighted. Major challenges faced with regard to these cheeses include limited standardisation, microbiological risks, and regulatory constraints. New strategies, such as selecting elite cardoon genotypes, developing tailored cultures, and adopting risk-based approaches, are discussed to reconcile authenticity with improved consistency, safety, and market competitiveness.

Table 1 summarises the characteristics of Iberian small-ruminant milk cheeses. Most of these PDO cheeses are produced from raw ewe’s or goat’s milk and coagulated with vegetable rennet derived from cardoon (*Cynara* spp.), typically without the addition of starter or adjunct cultures (non-starter cultures). Aqueous cardoon extracts are prepared from dried cardoon flowers (*Cynara cardunculus*) harvested at different maturity stages and from diverse regions. However, variation in drying methods, the inclusion of different parts of the plant, or mixing with other *Cynara* species (i.e., *Cynara scolymus* or *Cynara humilis*) result in significant differences in enzymatic activity [10]. Additional sources of variability in cheese characteristics, such as colour, aroma, texture, flavour and firmness, arise from milk origin, manufacturing practices, moisture content, ripening conditions, and the presence of indigenous microbial communities (yeasts, moulds, and bacteria).

### 1.2. Physicochemical and Biochemical Features Occurring During Cheese Ripening

Cheese ripening involves a complex series of biochemical reactions that shape its characteristic taste, aroma, and texture. Proteolysis is central in these transformations and results from the combined action of residual coagulant, indigenous milk enzymes, starter and non-starter microbiota, and, in some varieties, enzymes from secondary flora. The extent of protein, fat, and carbohydrate degradation is governed by intrinsic cheese properties and environmental conditions, mediated through microbial and enzymatic activity [13,14].

Lactic acid bacteria (LAB) are central to cheese ripening through lactose fermentation, proteolysis, and secondary catabolic reactions, contributing to the organoleptic characteristics of cheeses. The conversion of lactose to lactic acid reduces the pH, thereby shaping microbial dynamics and inhibiting foodborne pathogens. The final pH depends on the type of cheese produced and ripening conditions. In summary, the pH of rennet-coagulated milk cheeses varies between 5.2 and 4.8, while in cheeses produced by acid coagulation, it presents lower values (4.6–4.4). Additionally, the metabolism of citrate and of residual lactose generates key volatile compounds such as diacetyl and acetic acid.

Proteolysis is the primary driver of cheese matrix transformation. It leads to the progressive breakdown of caseins, resulting in texture softening and reduced water activity (a_w_) due to water binding by liberated amino and carboxyl groups. Concurrent pH changes enhance the release of sapid compounds during mastication. While these enzymatic breakdowns are essential for the characteristic flavour development resulting from the formation of peptides and free amino acids (FAAs), they also serve as substrates for secondary catabolic pathways (e.g., transamination and decarboxylation), contributing to both desirable aromas and potential defects, such as bitterness. Additionally, a significant safety concern in this process is the potential accumulation of biogenic amines. Excessive concentrations of these metabolites can pose health risks to the nervous and gastrointestinal systems. A recent review presents levels of several BAs in some cheeses. As an example, *Pecorino*, an Italian ewe’s milk cheese, presented levels of histamine, tyramine, putrescine and cadaverine of 65.5, 136.4, 100 and 120 mg/kg, respectively [15]. According to these authors, the limited published data suggest an acute reference dose of 50 mg of histamine for healthy individuals. In comparison, risk assessments indicate that while the acute lethal dose of tyramine for non-medicated individuals is 600 mg, the safety threshold drops drastically to just 6 mg for users of monoamine oxidase inhibitors. Raw milk cheeses represent a primary risk carrier for tyramine accumulation; they typically exhibit higher BA levels than pasteurised alternatives due to their complex microbial communities and elevated decarboxylase enzyme activity. Furthermore, while other BAs like putrescine and cadaverine possess low individual toxicity, they can synergistically amplify the toxic effects of both histamine and tyramine [15].

The extent of proteolysis is typically assessed through nitrogen fractions, including water-soluble nitrogen (WSN), trichloroacetic acid-soluble nitrogen (TCASN), and phosphotungstic acid-soluble nitrogen (PTASN). TCASN is often used as an indicator of ripening intensity. Proteolytic activity from coagulants, microbial enzymes, and indigenous milk proteinases releases peptides and FAAs, shaping cheese characteristics. Primary proteolysis (casein breakdown) dominates early ripening, affecting texture, while secondary proteolysis produces low-molecular-weight peptides (<500 Da) and FAAs that directly contribute to taste and serve as substrates for further metabolism [16]. FAA profiles vary according to cheese technology, microbial composition, and ripening conditions [17]. Specific amino acids contribute to distinct sensory attributes, such as bitterness (arginine) or sweetness (proline, serine, asparagine) [18]. LAB enzymatic systems further hydrolyse peptides, enhancing flavour complexity [19]. Texture evolution also influences the release of volatile compounds during consumption.

Lipolysis also plays a decisive role in flavour formation. Free fatty acids (FFAs) contribute directly to cheese aroma and act as precursors of carbonyl compounds, alcohols, alkanes, and esters. Lipases from milk and microorganisms, involved in cheese ripening, release short-chain fatty acids (SCFAs), which have a direct impact on flavour, while medium-chain fatty acids (MCFAs) and long-chain fatty acids (LCFAs) contribute less. Fernández-García et al. [20] reported significant differences in the lipolysis pattern among Spanish PDO cheeses made with raw ewes’ milk (*Manchego*, *Zamorano*, and *La Serena*). FFA concentrations were observed to increase with ripening, except for MCFAs (C10:0 to C14:0). The relative proportions of short-, medium-, and long-chain FFAs distinguished cheese types and production seasons. Discriminant analysis correctly classified 84% of *Manchego*, 93% of *La Serena* and 92% of *Zamorano* cheeses in each variety. These authors also reported that seasonality markedly influenced FFA levels, which were highest in spring cheeses. SCFAs (C4:0 to C8:0) accounted for 16.9%, 13.0%, and 15.8% of the total FFAs in *Manchego*, *La Serena*, and *Zamorano* cheeses, respectively, while unsaturated fatty acids (C18:1 and C18:2) contributed 27.0%, 34.5%, and 27.8%, respectively. Notably, *La Serena* cheese, despite its shorter ripening period, showed higher lipolysis and unsaturated fatty acid content, possibly linked to the ewe breed.

Tavaria et al. [21] showed that cheeses coagulated with vegetable rennet contained higher levels of volatile fatty acids (VFAs) than those coagulated with animal rennet, particularly acetic, propionic, isobutyric, butyric, decanoic, and dodecanoic acids. The differences in the amounts of isovaleric, hexanoic, and octanoic acids were not significant between coagulants. According to the authors, the stronger proteolytic activity promoted by vegetable rennet likely facilitates lipolysis (increasing VFA levels) by reducing the proteinaceous fraction of the curd. Other studies reported variable FFA patterns, sometimes decreasing over ripening, which was attributed to ester formation [22,23].

Overall, proteolysis and lipolysis should be considered as interconnected processes within a broader metabolic network that underpins cheese ripening. In addition to their sensory implications, these pathways contribute to the generation of bioactive compounds, including peptides and other metabolites released over ripening, largely through LAB activity, conferring potential health-related functionalities [24].

### 1.3. Milk-Clotting Agent (Cardoon)

About 75% of cheese varieties are produced through enzymatic coagulation using rennet, while alternative coagulants, mainly of plant origin, are employed in certain cheese types. These coagulants are used in the production of regional cheeses, often based on small ruminants’ milk. Proteolysis during cheese ripening, including the extent and nature of the degradation products, depends on: (1) endogenous milk proteinases (e.g., plasmin); (2) the proteinases present in milk-clotting agents; (3) proteolytic enzymes from LAB; (4) the type of cheese; and (5) the ripening conditions. These conditions include not only the milk origin but also geographical and technological factors, all of which modulate proteolytic patterns [25].

In Portugal, eight PDO cheeses (*Azeitão*, *Évora*, *Castelo Branco*, *Serra da Estrela*, *Nisa*, *Serpa*, *Amarelo Beira Baixa*, and *Picante da Beira Baixa*) and one PGI cheese (*Mestiço da Tolosa*) are produced using crude extracts from dried flowers of *C. cardunculus*. Similarly, Spanish PDO cheeses, namely, *Torta del Casar*, *La Serena*, *Los Pedroches,* and *Flor de Guía*, are also manufactured with *Cynara* spp. extracts. These cheeses are distinguished by the type of milk (predominantly ewe’s from local breeds), the use of vegetable rennet (*Cynara* spp. extracts), and the microbial communities of raw milk, which result in unique products. A characteristic feature of these cheeses is their soft paste with a slightly bitter or spicy flavour, largely attributed to the intense casein proteolysis induced by cardoon enzymes.

Extensive research has focused on the biochemical characterisation of the milk-clotting enzymes from cardoon flowers, their specificity towards caseins, and their technological implications in cheesemaking [10,26,27,28,29,30,31,32,33,34,35]. More recently, Gostin and Waisundara [36] highlighted the relevance of *C. cardunculus* flowers, covering aspects such as historical use, processing, preservation methods, regulatory considerations, and potential health benefits demonstrated by in vivo and in vitro studies, due to their hepatoprotective, anticarcinogenic, and hypocholesterolaemia functions.

Traditionally, cardoon extracts are prepared from dried flowers of *C. cardunculus*. However, extracts from *C. humilis* and *C. scolymus* have also shown effective milk-clotting activity [37,38]. Efforts have been undertaken to develop standardised formulations or to express *Cynara* spp. aspartic proteases (APs) in heterologous systems for recombinant production. Distinct proteinases with different clotting capacities have been found in *C. humilis* and *C. cardunculus*, the latter containing a chymosin-like enzyme [39], whereas *C. scolymus* produces three active proteases with milk-clotting activity (cynarases A, B, and C). Preservation studies indicate that filtered cardoon extracts can be stabilised for at least two months at room temperature with benzoic or sorbic acid, while unfiltered extracts require storage at 4 °C to minimise microbial contamination [40]. The authors stated that the extract treated with benzoic acid (1%) showed the best results in terms of bacterial contamination.

Regarding proteolytic activity, *C. cardunculus* and *C. scolymus* hydrolysed αs1-casein extensively (78% and 68%, respectively), whereas κ-casein degradation reaches 70% for *C. scolymus*, 57% for *C. humilis*, and 70% for *C. cardunculus* [34]. Variability in the activity of plant extracts results from the expression of different members of a multigene family of APs, associated with factors such as ecotype, flower part used, maturation stage, and moisture content [41,42]. Two major AP groups have been isolated from *C. cardunculus*: cyprosins and cardosins. Cyprosins are heterodimeric glycoproteins with different proteolytic and milk-clotting activities; notably, cyprosin 3 exhibits high proteolytic activity, with milk-clotting activity comparable to chymosin during early hydrolysis [43,44].

Table 2 and Table 3 summarise the main APs present in three *Cynara* species and the specific amino acid bonds cleaved in ovine and caprine caseins, respectively.

Among cardosins, A and B are the most abundant. Cardosin A displays narrower specificity and lower catalytic efficiency than cardosin B, but both preferentially cleave hydrophobic peptide bonds. Additional cardosins (E, F, G, and H) have also been described [43]. Cardosins promote clot formation by specifically cleaving the Phe_105_-Met_106_ bond of bovine κ-casein [46]. A comprehensive overview of APs’ structure, specificity, and technological applications, including recombinant strategies, has been provided by Alavi and Momen [47].

Considering the variation in AP in the different *Cynara* spp. and that, in several cases, not only *Cynara cardunculus* is used, a significative variation in the clotting properties of milk can occur. In addition, the use of different *Cynara* species (e.g., *Cynara scolymus*) or mixtures of *Cynara* species used in different proportions can lead to significant variations in enzymatic activity, leading to the appearance of different flavour compounds and batch-to-batch variability. Hence, despite its traditional value, *Cynara* spp. rennet poses several challenges for industrial application, namely, enzymatic and microbiological variability, the limited availability of flowers, and the risk of microbial contamination of aqueous extracts, which compromise its consistency and large-scale use. Aqueous extracts of cardoon flowers exhibit inconsistent milk-clotting activity and may introduce undesirable microorganisms into raw milk, raising concerns about safety and quality.

Ordiales et al. [10] reported that the intense and persistent flavour, together with a pungent, astringent, and bitter taste, as well as textural attributes such as smoothness and spreadability, are partly associated with the non-specific proteolytic activity of this vegetable rennet. Nevertheless, microbial proteolytic activity also contributes significantly to these sensory outcomes of cheeses. Furthermore, natural variability in the flower’s protease content, influenced by genetic differences and seasonal climatic conditions, leads to fluctuations in the organoleptic characteristics of the resulting cheeses. According to these authors, the pronounced heterogeneity of rennet employed in the manufacture of *Torta del Casar* cheese negatively impacts cheese quality, resulting in reduced yield and inconsistencies in key sensory factors across batches, which may translate into significant economic losses.

Although it is responsible for features of the cheeses, the use of cardoon extracts typically results in lower cheese yields compared to the use of animal rennet. Moreover, excessive proteolysis often leads to bitterness, mainly due to the release of hydrophobic peptides rich in aromatic amino acids. Additionally, the excessive bitterness associated with vegetable coagulant may pose some constraints to consumers’ acceptance [45]. As an alternative to the traditional crude aqueous extract of *C. cardunculus*, which has been reported to exhibit poor microbiological quality [29], a powdered vegetable coagulant (PVC) derived from these extracts was developed, patented, and successfully tested in the production of *Los Pedroches* and other Spanish cheeses. The authors reported that this PVC offers several advantages: it is free of viable microorganisms, is readily soluble in water or milk, is stable when stored in airtight containers without the need for preservatives and is easier to standardise and handle when compared to crude extracts. Tejada et al. [48] evaluated the sensory characteristics of *Los Pedroches* cheese made with animal rennet and two types of *C. cardunculus*-based coagulant (PVC and crude aqueous extracts). Cheeses produced with animal rennet were characterised by lower odour intensity, namely, pungent and acidic notes, a milder acid taste, and a slightly lighter colour. They also exhibited greater hardness and lower creaminess relative to cheeses made with vegetable coagulants. By contrast, cheeses produced with PVC showed similar organoleptic characteristics to those obtained with crude aqueous extracts. Differences became evident only after 90 days of ripening, when cheeses made with vegetable coagulant displayed a slightly more bitter taste than those made with animal rennet. Overall, ripening time was associated with an increase in most sensory scores, regardless of the coagulant used. Complementary findings were reported by Sanjuán et al. [43], who compared the physicochemical profiles of *Los Pedroches* cheeses produced with different rennet types over 100 days of ripening. The type of coagulant significantly influenced several parameters; cheeses made with animal rennet exhibited higher moisture, protein, and a_w_, whereas those made with vegetable coagulants contained higher fat and soluble nitrogen (SN). During ripening, common trends included increases in lactic acid content, ash, sodium chloride, and nitrogen fractions and a decrease in moisture, lactose, pH and a_w_.

Tejada et al. [49] investigated the compositional characteristics of *Murcia al Vino* goat’s cheese made with animal rennet and PVC. They reported that only pH, titratable acidity, ash, and calcium content were significantly affected by the type of coagulant. By contrast, esterified fatty acids were not significantly affected by the coagulant type or ripening time. The predominant esterified fatty acids in this cheese were C16 and C18:1. Furthermore, Tejada et al. [50] investigated primary and secondary proteolysis, as well as changes in hydrophilic and hydrophobic peptides, during the ripening of *Murcia al Vino* goats’ milk cheese produced with PVC or with calf rennet. After 60 days, cheeses made with calf rennet presented values of 19.04% water-soluble nitrogen (WSN), 9.61% non-protein nitrogen (NPN), 0.55% ammonia nitrogen (N–NH_3_), and 0.66% amino acid nitrogen (AAN). By contrast, *Murcia al Vino* cheese produced with PVC showed intense proteolysis with corresponding values of 34.79%, 11.16%, 0.59% and 0.88%, respectively. The degradation products of β-CN (γ-CN fractions) were higher in cheeses manufactured with animal rennet, whereas cheeses made with PVC contained higher concentrations of hydrophilic and hydrophobic peptides, as well as a higher hydrophobic/hydrophilic peptides ratio. Tejada et al. [51] also compared *Los Pedroches* cheese made with PVC versus animal rennet. No differences were observed between the two types of coagulants in most chemical parameters or in the main microbial groups evaluated (total viable microorganisms, LAB, Enterobacteriaceae, coliforms, *Escherichia coli*, moulds and yeasts). However, cheeses produced with PVC showed higher casein hydrolysis after only two days of ripening, with increased levels of SN, NPN, AAN and N-NH_3_, as well as higher flavour and aroma scores, compared to animal rennet cheeses. Gálan et al. [52] compared different concentrations of PVC with calf rennet in ewe’s milk cheese, analysing chemical, biochemical, and sensory characteristics over six months of ripening. Most chemical parameters did not differ significantly between the coagulant types. However, cheeses produced with twice the amount of PVC showed markedly higher levels of casein hydrolysis, measured as SN, NPN, AAN, and N-NH_3_ after only two days of ripening, compared to cheeses made with the standard PVC dose. In addition, only SN and NPN levels were significantly higher in PVC cheeses than in those made with calf rennet. Sensory analysis indicated that cheeses produced with PVC showed enhanced organoleptic characteristics relative to those made with calf rennet. The bitter taste of cheeses with a doubled concentration of PVC was not significantly stronger than that of cheeses with PVC. The authors concluded that the higher proteolytic activity of PVC enzymes enables the production of fully ripened cheeses with the desired sensory properties approximately three months earlier than with calf rennet. Prados et al. [53] compared PVC with calf rennet in *Manchego* cheese over a six-month ripening period by assessing chemical, biochemical, and sensory characteristics. No significant differences were observed for most chemical parameters. However, cheeses produced with PVC showed higher casein hydrolysis, with significantly higher SN levels, while other nitrogen fractions were only slightly elevated. Sensory evaluation indicated that cheeses made with PVC had a superior odour, colour, taste intensity, and creaminess compared to those made with animal rennet.

Pino et al. [17] investigated *C. cardunculus* coagulant vs. calf rennet in traditional raw goats’ milk cheese, focusing on FAA profiles during ripening. Principal component analysis (PCA) of FAA data revealed that variability could be explained by two PCs: PC1, associated with leucine, valine, lysine, glycine, tyrosine and aspartic acid, separated cheeses according to ripening time (2 to 60 days versus 90 to 120 days); and PC2, correlated with arginine, histidine, tryptophan, serine, and threonine, distinguished cheeses according to the type of coagulant used.

Cardoon flower availability is another critical issue. In the *Serra da Estrela* PDO region, annual cheese production requires over one ton of cardoon flowers, which underscores the need for standardised raw materials [41]. To address this problem, researchers evaluated the biodiversity of 12 cardoon cultivars over three harvest seasons using 34 morphological descriptors. Significant variation was observed in 24 morphological characteristics, reflecting wide genetic diversity [42]. Moreover, biochemical variability in cardosin profiles highlighted the necessity of characterising cardoon germplasm based on morphological, biochemical, and genetic parameters. The authors explained that propagation via plant achenes does not guarantee genotypic stability, recommending in vitro or vegetative propagation to produce selected cardoon genotypes with consistent biochemical and morphological characteristics. Such approaches would enable breeding programs to generate cardoon plants optimised for flower production [41].

Gomes et al. [54] addressed the technological variability of 15 cardoon flower ecotypes from the Portuguese *Alentejo* region, assessing milk-clotting activity, coagulation properties, and cheesemaking yield. Milk-clotting activity ranged from 57 to 128 milk-clotting units (MCUs)/g. Coagulation parameters also showed wide variation: clotting time (R) ranged from 779 to 1207 s; aggregation speed (K_20_) ranged from 640 to 1619 s; and gel firmness (A_2R_) ranged from 2.47 to 4.65 mm. Cheese yields varied between 22.8 and 28.9 g curd (db)/100 g milk. Multivariate analysis confirmed significant variability among ecotypes, grouping them into five clusters primarily influenced by milk-clotting activity, gel firmness, and micellar aggregation rate, followed by proteolytic activity. The authors emphasised that such variability could affect the final cheese properties.

Silva et al. [38] evaluated the impact of various wild *C. cardunculus* populations on the cheesemaking process of *Évora*, *Serpa*, and *Nisa* Portuguese PDO cheeses. Using Urea-PAGE (Polyacrylamide Gel Electrophoresis), the researchers mapped the breakdown of casein fractions into four distinct zones: β-caseins, αS-caseins, and their respective degradation products, γ-caseins and pre-αS-caseins. The study identified significant statistical relationships between the primary caseins and their breakdown products across all three cheese types. A high coefficient of determination (r^2^ > 0.80) was found between αS-casein and its degradation into pre-αS-casein. By contrast, the correlation between β-casein and γ-casein remained below 0.80. These results suggested that the degradation of αS-caseins serves as a highly reliable indicator of the extent of proteolysis. Monitoring this specific fraction provides producers and researchers with a viable tool to measure and standardise the ripening process for *Évora*, *Serpa*, and *Nisa* cheeses, ensuring consistent quality across these geographically distinct PDO products.

As referred, the industrial application of these plant-based coagulants has been hindered by a lack of standardisation of *C. cardunculus* extracts [44]. Recent research has sought to overcome these hurdles by developing standardised native formulations and the heterologous production of synthetic enzymes like cardosins and cyprosins (see Section 3). Furthermore, the use of artichoke (*C. scolymus*) has emerged as a promising industrial alternative due to its high availability and coagulant potential [55].

Recent insights on the use of *Cynara* spp. as a milk-clotting agent have been reported by Nicosia et al. [56], Bande-De León et al. [57], and Ritota et al. [58].

Finally, Rana et al. [59] analysed the trends in the environmental sustainability of the *C. cardunculus* supply chain. The research outlined the importance of biomass and waste valorisation for energy and materials, yield optimisation, environmental impact analysis, and irrigation management. It also examined strategic responses to climate change and resource scarcity, emphasising the link between circular economy principles and environmental metrics. Through a comprehensive look at the *C. cardunculus* supply chain, the paper identified emerging research gaps in both the cultivation and processing phases.

Ultimately, understanding the interplay between enzymatic activity, microbial communities, and technological treatments is vital for preserving the unique heritage and consumer value of PDO cheeses in a modern industrial context [60].

### 1.4. Raw Milk and Cheese Microbiota

The use of raw milk enables its indigenous microbiota to play a fundamental role during cheese ripening. Bacteria, yeasts, and moulds contribute directly through metabolic activity, or indirectly via the release of intracellular enzymes, following cell lysis. The complex microbial communities and their dynamics during cheese production and ripening are key determinants of food safety and sensorial quality [61]. Raw milk cheeses are generally dominated by LAB (e.g., *Lactococcus* spp., *Lactobacillus* spp., and *Enterococcus* spp.); however, other microorganisms, such as Enterobacteriaceae, coliforms, *Staphylococcus* spp., *Pseudomonas* spp., and *Listeria* spp., may also be present [62]. In cheeses without added starters, a limited number of LAB genera usually prevail, playing critical roles in the development of organoleptic properties of artisanal cheeses [63,64]. Cheese microbiota is commonly classified into starters and secondary flora (non-starter), comprising bacteria and fungi that further contribute to the development of organoleptic properties. Although LAB dominate, Gram-positive catalase-positive bacteria, yeasts, moulds, and Gram-negative bacteria (e.g., *Citrobacter* spp., *Enterobacter* spp., *Pseudomonas* spp.) may also form subdominant or even codominant populations [65].

LAB play central roles in proteolysis through their cell-envelope proteinases, breaking down caseins into oligopeptides, which are further degraded into shorter peptides and amino acids by intracellular peptidases. These peptides, amino acids, and their derivatives not only influence texture and flavour but also generate bioactive molecules with reported antioxidant, antimicrobial, anti-inflammatory, immunomodulatory, analgesic/opioid, inhibition of the angiotensin-converting enzyme, and antiproliferative activities. Some LAB also synthesise functional lipids (e.g., conjugated linoleic acid) with anti-inflammatory and anticarcinogenic activity, vitamins, and bacteriocins or can release γ-aminobutyric acid (GABA), a non-protein amino acid that participates in physiological functions, such as neurotransmission and hypotension induction. Several other bioactive compounds are found in cheese (e.g., peptides, exopolysaccharides, fatty acids, organic acids, vitamins), all of which exhibit biological activity [24].

LAB dominate the microbiota of Portuguese PDO cheeses such as *Serra da Estrela*, *Azeitão*, *Évora*, *Serpa*, and *Picante da Beira Baixa*, as well as Spanish cheeses, including *Torta del Casar*, *La Serena*, and *Los Pedroches* [10,20,66,67,68,69]. As an example, *Lactobacillus paracasei* subsp. *paracasei*, *Leuconostoc mesenteroides* subsp. *mesenteroides*, *Lactococcus lactis* subsp. *lactis* and *Enterococcus faecium*, isolated from *Serra da Estrela* cheese, exhibited distinct peptidase profiles (dipeptidyl aminopeptidase, endopeptidase, dipeptidase and carboxypeptidase activities), with the crude free-cell extracts (CFEs) of *L. paracasei* showing the highest aminopeptidase activity (followed by the CFEs of *L. mesenteroides* and of *L. lactis*), preferentially cleaving peptide bonds between hydrophobic or basic amino acids, which are important for flavour development [19]. Esterase activities from wild *L. lactis* and *Lactobacillus plantarum* strains isolated from *Serra da Estrela* cheese also contribute to lipolysis, leading to FFA release during ripening, with *L. lactis* showing greater activity towards SCFA derivatives [69]. Moreover, *L. plantarum* was shown to produce a distinct metallo-aminopeptidase with potential debittering activity, which supports its inclusion in starter formulations [70].

Tavaria et al. [16] reported that the highest daily increase in FAAs during *Serra da Estrela* cheese ripening occurred between the 7th and the 18th days (6.1%), the 60th and the 90th days (2.0%) and between the 150th and the 180th days (2.0%), coinciding with the exponential growth of LAB, as formerly reported by Dahl et al. [69]. These findings suggest that the vegetable coagulant itself has limited peptidase activity, confirming that peptidases synthesised by adventitious microorganisms naturally present in this cheese are the main contributors to FAAs’ release. In a complementary study, Tavaria and Malcata [70] evaluated LAB strains isolated from *Serra da Estrela* cheese, namely, *L. paracasei* ssp. *paracasei*, *L. mesenteroides* ssp. *dextranicum*, *L. lactis* ssp. *lactis* and *E. faecium*, for aminotransferase, oxidase, and dehydrogenase activities against five FAAs. Such enzymatic conversion of FAAs is central to the synthesis of flavour compounds throughout ripening. Methionine, one of the key amino acid precursors of sulphur volatile compounds and extensively catabolised by the studied strains (especially *E. faecium*), was the most rapidly and extensively degraded, which suggests the suitability of these species for inclusion as starters.

*Enterococcus* spp. are often linked with dairy products due to their lipolytic, proteolytic, and citrate-metabolising activities, which contribute to flavour and aroma. They are also known producers of bacteriocins with an inhibitory spectrum against foodborne pathogens dominant in raw milk cheeses, such as *Listeria* spp. However, excessive enterococcal growth may negatively affect cheese quality, and their proteolytic activity compared to other LAB may accelerate ripening but also increase the risk of off-flavours.

Flavour contributions also arise from esterases, which hydrolyse milk fat, and from citrate breakdown, yielding acetaldehyde, acetoin, and diacetyl. Even so, given their potential pathogenicity and capacity to harbour transferable antibiotic resistance genes, only carefully selected strains of *Enterococcus* spp. should be considered for food industry applications [71].

Chaves-López et al. [72] highlighted the role of Enterobacteriaceae isolated from *Pecorino* cheese (Italian PDO cheese), showing that many strains presented physiological activities that influenced flavour, even with non-viable cells. Their enzymes contribute to taste, aroma, and texture development, which are closely correlated to Enterobacteriaceae counts [72,73]. Lactose and citrate co-metabolism previously reported for *L. lactis*, *Leuconostoc* spp., and *Enterococcus faecalis* [74] also occurs in Enterobacteriaceae, producing aromatic compounds. While their fatty acid metabolism generates precursors of methyl ketones, alcohols, lactones, and esters, they may accumulate substances that negatively impact flavour, such as butyric acid [75].

Despite the advantages of the use of raw milk for the sensory characteristics of cheeses, its use can cause variability in organoleptic and overall quality due to the uncontrolled activity of wild microbiota, while also raising food safety concerns. Van den Brom et al. [75] reported that in several regions, sheep and goat dairy serves as a vital alternative for children and individuals with bovine milk allergies. Additionally, in areas that are geographically unsuitable for cattle, sheep milk remains a staple of the daily diet. However, the authors reported that the consumption of raw milk and its derivatives carries significant zoonotic risks. Key pathogens associated with sheep milk include *Brucella melitensis*, *Campylobacter* spp., *Coxiella burnetii*, *Leptospira* spp. *Listeria* spp., *Salmonella* spp., Shiga-toxin producing *E. coli*, tick-borne encephalitis, *Toxoplasma gondii*, and Rift Valley fever. These risks can be mitigated through robust flock health management, hygienic milking procedures, and controlled processing and storage. Small-scale systems often present a higher risk profile than industrialised systems due to their less standardised protocols. Vulnerable populations, specifically the young, old, pregnant, and immunocompromised, should exercise caution regarding raw dairy. Consequently, strict hygiene and rigorous periodic testing across the supply chain are essential.

This has prompted calls for hygienic practices and the adoption of defined starter cultures to improve both consistency and safety. Among the most relevant safety issues is the potential survival of *Listeria monocytogenes* in raw milk cheeses. Regulation (EC) N°1441/2007 [76] distinguishes cheeses in which *L. monocytogenes* can, or cannot, survive based on intrinsic properties such as pH and a_w_. Therefore, risk assessment must consider determinants of this growth/no-growth pattern according to cheese type. Soft, semi-soft, and non-acidic fresh cheeses present a high risk of supporting *L. monocytogenes* growth, whereas growth is generally inhibited in hard, semi-hard, and acidic fresh cheeses due to their lower pH and/or moisture content. Gérard et al. [77] reported no significant differences in the prevalence of *L. monocytogenes* between raw and pasteurised milk cheeses, with contamination more often linked to inadequate hygiene during post-pasteurisation or post-processing steps. Hard and semi-hard cheeses, characterised by a_w_ values below 0.97, are considered less favourable environments for *L. monocytogenes* growth compared to fresh, soft, and semi-soft cheeses [76]. Supporting these findings, studies on the prevalence of *Listeria* spp. in Portuguese soft cheeses made from raw ewe’s milk revealed that 75% were contaminated with *Listeria* spp. *L. monocytogenes* was present in 46% and *Listeria innocua* in 29%, though propagation of *L. monocytogenes* would depend on factors such as organic acid concentration (influencing pH) and salts (influencing a_w_), as well as the storage temperature [78]. Considering regional cheese types (e.g., *Castelo Branco*, *Amarelo da Beira Baixa*, and *Picante da Beira Baixa*), only the latter appears not to pose a risk, due to its high salt content and low a_w_.

*Staphylococcus aureus* has also been frequently detected in raw milk cheeses, often at higher incidence than other foodborne pathogens [65]. Although this microorganism can produce enterotoxins associated with foodborne illness, only high cell densities lead to toxin production at levels hazardous to public health [79].

In addition, mould growth in cheese presents both quality and food safety issues, leading to significant economic losses. Certain moulds may also produce mycotoxins, further elevating the risk. Márin et al. [80] identified *Geotrichum* and *Fusarium* as the most common genera in milk samples and in one-month ripened *Manchego* cheese, suggesting direct transfer from raw milk. By contrast, the mycobiota of long-ripened cheeses was dominated by *Penicillium* spp., originating primarily from the environment of the ripening chambers. This highlights the role of the dairy plant’s internal air mycobiota as a major contamination source. Moreover, the fungal diversity in ovine milk varied across farms, which indicates that airborne transfer from the farm environment can directly impact cheese quality.

The growth of moulds on the surface of traditional cheeses is typically controlled through labour-intensive washing procedures, because PDO specifications generally prohibit the use of preservatives such as natamycin. Preventing visible mould growth is essential because it compromises product quality. The application of protective cultures to the cheese surface has been proposed as a potential strategy to minimise this problem, offering both economic and safety benefits. Nevertheless, the implementation of this approach remains limited, as PDO regulations generally restrict such practices.

It should be noted that, although foodborne outbreaks associated with dairy products are relatively rare, those reported have typically been associated with the use of unpasteurised or improperly pasteurised milk and/or post-contamination. This fact presents a challenge for the commercialisation of raw milk cheeses in international markets with stricter safety regulations [81].

Table 4 presents some components resulting from enzymatic and microbial action during cheese ripening that have a significant role in cheese flavour. The list is associated with some Iberian cheeses. However, some of these components (e.g., some SCFAs or FAAs) may be found in all the cheeses listed in Table 1.

## 2. Specific Features of Spanish and Portuguese PDO Cheeses Coagulated with Cardoon

The production of PDO cheeses in Spain and Portugal is closely linked to areas with significant small-ruminant milk production. While certain cheeses such as *Manchego* and *Serra da Estrela* have been extensively studied, others remain poorly documented.

Freitas and Malcata [88] reviewed the microbiology and biochemistry of Iberian cheeses made with ovine and caprine milks. Since then, no other report has focused on Iberian small-ruminant milk cheeses, although some reviews on Portuguese or Spanish PDO cheeses have been published.

### 2.1. Spanish Small Ruminants’ Milk PDO Cheeses

Table 1 summarises Spanish PDO cheeses produced with ewe’s and/or goat’s milk coagulated by cardoon. Spain produces approximately 76 thousand tons of ewe’s milk cheeses annually, of which PDO *Manchego* cheese represents ca. 25%. By contrast, *La Serena* and *Torta del Casar* together account for less than 500 tons [89]. The production of *Flor de Guía* cheese was reported to be below 1 ton in 2010 [90]. These figures highlight the dominant role of *Manchego* cheese in Spanish ewe’s milk cheese production, while other PDO cheeses contribute less than 1%. Overall, in Spain, of the nearly 300 thousand tons of cheese produced annually from ewe, goat, or mixed milks, fewer than 10% correspond to PDO cheeses.

#### 2.1.1. Flor de Guía

*Flor de Guía* is a Spanish PDO cheese produced on the island of Gran Canaria. It is classified as a full-fat or half-fat cheese made from Canarian ewe’s milk, which may be blended with milk from Canarian cows and/or goats. The cheese typically measures 4–8 cm in height and 20–30 cm wide, and its weight ranges from 2 to 5 kg. Its name derives from a region in northern Gran Canaria called *Santa María de Guía* and from the term ‘*flor*’, referring to the juice extracted from the flower of cardoon and globe artichoke species used to curdle the milk. Although kid rennet may also be used, the PDO regulations for *Flor de Guía* cheese specify two essential requirements: the exclusive use of vegetable rennet from *C. cardunculus* var. *ferocissima* or *C. scolymus* as the milk-clotting agent, and strict proportions of milk according to the species of origin. Ewe’s milk must account for at least 60% of the mixture, while cow’s milk must not exceed 40%, and goat milk must not exceed 10%. Carrascosa et al. [91] investigated the effect of varying concentrations of cardoon extract on the coagulation of different types of milk and milk mixtures. Their results demonstrated that coagulant activity increased both with higher concentrations of cardoon extract and with a greater proportion of ewe’s milk in the mixtures used as substrates. Rincón et al. [92] evaluated the FFA composition and sensory profiles of cheeses produced from raw goat’s milk using a vegetable coagulant derived from *C. cardunculus*. For comparison, cheeses manufactured with a commercial coagulant, a traditional kid rennet paste, and a mixed coagulant (vegetable coagulant combined with kid rennet) were also analysed. The contents of SCFAs, MCFAs, and LCFAs varied according to both the type of coagulant and the ripening time. Cheeses produced with vegetable coagulants exhibited a significantly higher odour and flavour intensity than those made with commercial coagulants; however, these differences diminished as ripening progressed. Multivariate analysis enabled the discrimination of cheese samples according to ripening time based on their lipolytic profiles and according to coagulant type based on sensory attributes.

#### 2.1.2. La Serena

*La Serena* cheese is produced in the district of *La Serena* (*Extremadura*, Spain) from *Merino* ewe’s raw milk coagulated with *C. cardunculus*. The minimum ripening period is 60 days. When the cheese develops a soft, creamy centre, it is marketed as *Torta de la Serena*, whereas extended ripening yields a firmer cheese with a stronger flavour, sold as *Queso de la Serena*. *Torta de la Serena* differs from *Torta del Casar* cheese, also produced in *Extremadura*, because it is made exclusively with *Merino* ewe’s milk, while *Torta del Casar* can be made from mixed milk breeds, and it contains a lower proportion of cardoon extract in its formulation. These differences contribute to the softer texture of *Torta de la Serena*. Roa et al. [93] demonstrated that the milk clotting in *La Serena* cheese is mainly driven by the high proteolytic activity of the enzymes present in cardoon flowers. Approximately 27% of the total coagulant added to milk was retained in cheese, while 78% appeared in whey. The observed stability of vegetable rennet during cheese ripening was linked to a relatively constant composition of the cheese (moisture, pH, NaCl, fat, and protein). Electrophoretic analyses revealed that αS-casein was less susceptible to proteolysis than β-casein. Water-soluble nitrogen (WSN) increased only during the first 30 days of ripening, while the ratios of NPN/TN and of AAN/TN increased progressively with ripening time.

Fernández-García et al. [20] reported that the levels of all individual FFAs increase with aging in *La Serena* cheese. They also observed that the season of manufacture significantly influenced FFA concentration. Semi-cured cheeses produced in fall or spring and cured cheeses made in spring showed the highest FFA content compared to *Manchego* and *Zamorano* cheeses. The same authors also investigated the effect of the manufacturing season and ripening time on volatile terpenoids and benzenoids of plant origin in *La Serena* cheese during summer, winter, and spring. Terpenoids (mainly terpenes) were primarily derived from animal feed, whereas benzenoids originated mainly from *C. cardunculus* extract. During ripening, concentrations of γ-curcumene, α-humulene, ethyl benzene and propyl benzene and xylene isomers decreased, while those of α-terpineol, verbenone, benzyl alcohol, 2-phenyl-ethanol, benzoic acid methyl ester and the phenolic compounds increased significantly. Seasonal variation was also observed: safranal, geranyl acetone, γ-curcumene, and α-curcumene were more abundant in spring cheeses, while alkyl benzenes and other benzenoids were significantly more abundant in summer cheeses.

Historically, the microbiological quality of *La Serena* has been inconsistent, with high coliform counts reported [94]. Ordiales et al. [65] noted that autochthonous microbiota can influence both the cheese texture and the suppression of pathogens. Merchán et al. [95] found that the unique microbiota of *La Serena* and *Torta del Casar* are shaped more by specific dairy practices than by the ripening process itself. While *L. lactis* dominates *Torta del Casar*, *L. mesenteroides* is more prominent in *La Serena*. Research by Merchán et al. [96] indicates that *Yarrowa alimentaria*, *Y. lipolytica*, *K. lactis*, and *Pichia fermentans* dominate the fungal populations in all samples of *La Serena* and *Torta del Casar*. Other species such as *Debaryomyces hansenii*, *Debaryomyces vindobonensis* and *Geotrichum candidum* were also identified in specific dairies. The authors reported that the PDO and the ripening time factors did not significantly influence the fungal diversity between different cheese samples, suggesting that minor changes in manufacturing practices between the types of cheeses have a negligible impact on mycobiota composition. Conversely, the dairy factor contributed to the variability of the fungal composition of each cheese type.

Del Pozo et al. [97] evaluated the changes in the microflora of *La Serena* ewes’ milk cheese during ripening and reported that Enterobacteriaceae decreased by approximately 5% from manufacture to 60 days of ripening, while LAB increased consistently throughout ripening. Their findings highlighted the variability inherent to artisanal cheesemaking and underscored the need for improved standardisation of manufacturing practices, particularly to ensure the control of potentially hazardous microbial groups in traditional cheeses.

#### 2.1.3. Los Pedroches

**Figure 1:**
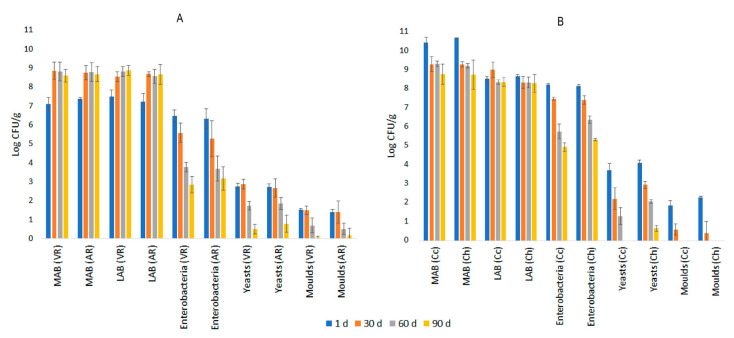
Microbial counts in *Los Pedroches* cheese: (**A**) cheeses made with vegetable rennet (VR) or animal rennet (AR); (**B**) cheeses made with *Cynara cardunculus* (Cc) or *Cynara humilis* (Ch). MAB: mesophilic aerobic bacteria; LAB: lactic acid bacteria (based on data from Tejada and Fernández-Salguero [51] and Vioque et al. [39]).

*Los Pedroches* cheese is manufactured in the northern region of *Andalucía*. It is a seasonal, hard-paste, high-fat ripened cheese made from raw *Merino* ewes’ milk. Coagulation is usually carried out by *C. cardunculus,* although *C. humilis* is often used. Sometimes, both species are combined. Tejada and Fernández-Salguero [51] reported higher microbial counts in cheeses produced with *C. cardunculus* compared to animal rennet (Figure 1A). Mesophilic aerobic bacteria (MAB) reached maximum counts of 8.6 log CFU/g after 90 days, irrespective of coagulant. Enterobacteria, coliforms, lactobacilli, moulds, and yeasts peaked between 2 and 8 days of ripening. Higher enterobacteria counts after 60–90 days in cheeses made with animal rennet were attributed to their higher pH, which favoured bacteria survival. Coliforms followed a similar trend. Mould counts reached a maximum by day 15 and decreased to <0.6 log CFU/g after 60 days of ripening, while yeast counts were comparable for both coagulants.

Vioque et al. [39] compared the chemical, microbial, and sensory characteristics of *Los Pedroches* cheese made with aqueous extracts of flowers of *C. cardunculus* or *C. humilis* throughout ripening. The evolution of the microbial groups is depicted in Figure 1B. The two cardoon species had no appreciable effect on cheese composition (moisture, fat, protein, NaCl), a_w_ or sensory attributes. However, cheeses produced with *C. humilis* exhibited a lower lactic acid content and higher pH values. Proteolysis was more pronounced and progressed faster in cheeses coagulated with *C. cardunculus*, as reflected by significantly higher levels of WSN and NPN, whereas AAN levels were similar for both coagulants. Microbial counts of MAB, coliform, and lactobacilli were similar between cheeses produced with either coagulant; however, cheeses made with *C. humilis* showed higher levels of enterobacteria, yeasts, and moulds. After two days of ripening, the total MAB counts in cheese were nearly 5 log units higher than in milk, and enterobacteria, coliforms, and lactobacilli were approximately 4 log units higher. These increases were attributed to microbial growth during coagulation, physical entrapment of microorganisms in the curd, and additional contamination introduced by aqueous cardoon extracts [29].

#### 2.1.4. Torta del Casar

*Torta del Casar* is a PDO cheese from the *Extremadura* region (*Cáceres*), produced from the raw milk of *Merino* and *Entrefino* ewes. It is characterised by a soft, spreadable texture, a slightly bitter taste, and a strong aroma. These attributes are largely associated with the use of the vegetable coagulant prepared as an aqueous extract from dried flowers of *C. cardunculus*. Ordiales et al. [10] evaluated the physicochemical, microbial, and sensory properties of *Torta del Casar* and correlated these parameters with the milk-clotting and proteolytic activities of the vegetable rennet used. Variations in rennet activity significantly influenced cheese composition during early ripening and affected texture and sensory attributes at the 60th day of maturation. Aqueous extracts of *C. cardunculus* exhibited high clotting activity after 24 h of maceration and limited β-casein degradation. They produced cheese with greater creaminess. The relative hydrolysis of k-casein and β-casein was shown to be the key determinant of texture properties, including firmness and shearing work [33].

**Figure 2:**
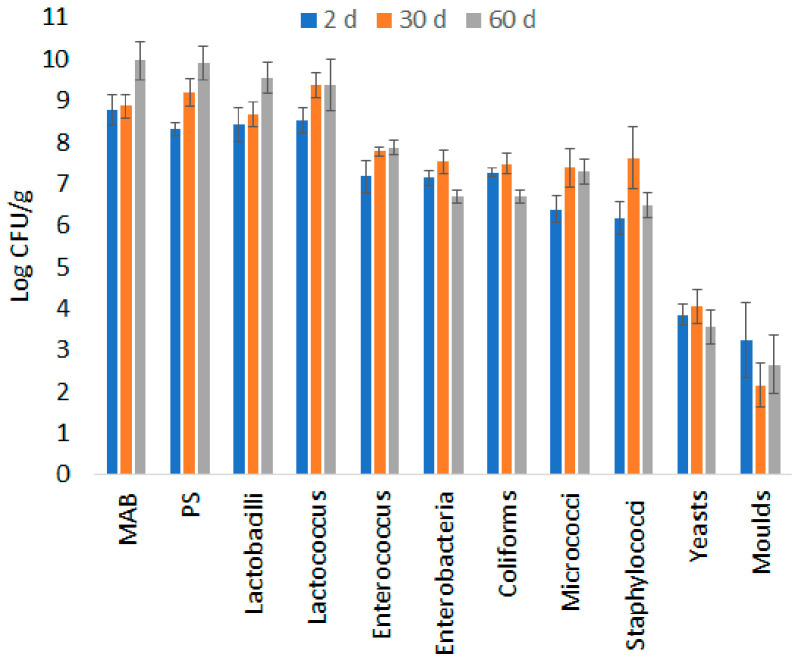
Bacterial groups of PDO *Torta del Casar* cheese (based on data from Ordiales et al. [64]). MAB: mesophilic aerobic bacteria; PS: psychrotrophic bacteria.

In a subsequent study, Ordiales et al. [64] investigated the dynamics of adventitious microbiota and foodborne pathogens during the ripening of *Torta del Casar* (Figure 2). Based on spreadability measurements, cheeses were classified into four clusters ranging from non-creamy to very creamy. Although total microbial counts did not differ significantly among groups, the composition of the microbiota differed markedly. The creamiest cheeses were associated with *L. lactis* subsp. *cremoris* and with the presence of *Serratia proteamaculans* and *Enterococcus devriesei*, whereas the less creamy cheeses exhibited distinct LAB and enterococcal profiles. Microbiological identification revealed that *Leuconostoc carnosum* and *Lactobacillus sakei* predominated in the least creamy cheeses, while *L. lactis* ssp. *cremoris* was mainly identified in the creamiest group. Other LAB, including *Lactococcus raffinolactis*, *Lactobacillus casei*, *Enterococcus durans*, and *E. devriesei*, were distributed across multiple groups. The predominance of *L. lactis* ssp. *cremoris* in the creamiest cheeses was attributed to its capacity to produce exopolysaccharides and proteolytic enzymes, which may enhance texture development. Additional microbial groups identified included *Staphylococcus saprophyticus* and *S. epidermidis,* with the latter more prevalent in creamy cheeses. *E. faecalis* dominated in the least creamy group. Overall, these findings highlight the central role of indigenous microbiota composition, rather than total microbial load, in determining the final texture and sensory properties of *Torta del Casar* cheese. *Listeria* spp. were identified at 30 days of ripening in all cheeses except for one group. However, no *Listeria* spp. were identified in any batch at the end of ripening (60 days). All isolates were identified as *Listeria ivanovii*, which suggests contamination from raw materials and highlights the importance of raw material quality. As *L. monocytogenes* is primarily an environmental pathogen, the main safety risk for *Torta del Casar* is environmental contamination during the manufacture, ripening or washing of cheeses. *Salmonella enterica* was identified only at early ripening stages and was absent at the end of ripening. *S. aureus* was found in all groups during early ripening but declined after salting, which inhibited its growth. Sulphite-reducing clostridia were identified in all groups, although counts were minimal after 30 days of ripening. Despite similar counts of LAB and enterobacteria among cheese groups, each group exhibited a distinct LAB composition, which appeared to influence both texture development and pathogen dynamics. Differences were most evident between the least creamy and the creamiest cheeses, particularly regarding the presence of *L. lactis* ssp. *cremoris* and *Enterococcus devriesei* among LAB and *Serratia proteamaculans* among enterobacteria in the creamiest group.

Ordiales et al. [65] further demonstrated that, although LAB play a dominant role in fermentation, other microbial groups contribute to the characteristic flavour of *Torta del Casar* and help modulate excessive LAB metabolite accumulation. Organic acids were the most abundant volatile compounds, followed by alcohols and carbonyls. Acetic acid and several alcohols were associated with high LAB counts, whereas enterobacteria were linked to semi-volatile fatty acids. Gram-positive catalase-positive (GPCP) cocci correlated with esters and methyl ketones. Multivariate analysis confirmed strong associations between microbial populations and the volatile profile, particularly highlighting the contribution of enterobacteria to SCFAs and the role of GPCP cocci in ester (acetic acid ethyl ester, butanoic acid ethyl ester) and methyl ketones (2-heptanone and 2-nonanone) formation.

Correlation analyses between microbial populations and sensory attributes indicated that cheeses with high LAB counts, mainly *Lactobacillus* spp., scored higher for salty, sour, and bitter tastes, as well as for flavour intensity. The ewe’s milk flavour was positively correlated with GPCP cocci, while off-flavour perception was primarily associated with acetic acid concentration. Notably, over 60% of the variability in acetic acid levels could be explained by lactobacilli and GPCP cocci counts, with GPCP cocci showing a negative regulatory effect on acetic acid accumulation, thereby contributing positively to flavour balance.

In a related study, Ordiales et al. [33] reported consistently high total viable bacteria and LAB counts (>8 log CFU/g) throughout the ripening of *Torta del Casar*, with substantial variability during the early stages. GPCP cocci counts remained relatively constant at approximately 7 log CFU/g. No direct correlation was observed between coagulant activity and microbial counts, whereas textural attributes were strongly associated with the extensive proteolysis characteristic of cardoon-coagulated cheeses.

Lipolysis during the ripening of *Torta del Casar* cheese was further characterised by Delgado et al. [82], who reported a significant increase in FFAs, particularly SCFAs, which are key contributors to cheese aroma. Most FFAs increased during ripening, except for valeric (C5:0) and margaric (C17:0) acids. Isovaleric (iC5:0) and linolenic (C18:3) acids, absent at day 1, showed marked increases by the end of ripening. Acetic acid was the most abundant FFA at the end of maturation, but its occurrence was attributed mainly to microbial fermentation rather than to lipolysis. SCFAs, notably butyric, isobutyric, and isovaleric acids, increased substantially throughout ripening, whereas MCFAs remained relatively stable. LCFAs increased until the 60th day, with no further changes thereafter. Lipid oxidation increased during the first month and decreased in the later stages of maturation, without compromising cheese quality.

The unique sensory profiles of these cheeses are also dictated by their microbial ecology. The microbiota of *Torta del Casar* and *La Serena* are distinct, shaped more by specific dairy environments and manufacturing practices than by the ripening period itself [97]. Key LAB, such as *L. lactis* and *L. mesenteroides*, drive the ripening process, while the presence of specific autochthonous flora has been linked to variations in creaminess and the natural suppression of pathogens like *L. monocytogenes* [63].

Beltran Sanahuja et al. [61] compared PDO ewe’s milk cheeses with non-PDO counterparts. Non-PDO samples generally had higher moisture and a_w_. Conversely, PDO cheeses exhibited a higher ester content, a greater breaking force, and significantly higher consumer scores. This suggests that PDO cheeses are typically more mature when sold, which leads to superior sensory properties.

### 2.2. Portuguese Small Ruminants’ Milk PDO and PGI Cheeses

Portuguese PDO and PGI cheeses produced from ewe’s and/or goat’s milk and coagulated with cardoon are indicated in Table 1. Of the six thousand tons of ewe’s milk cheese produced annually in Portugal, PDO cheeses account for less than 10%. Regarding cheeses manufactured from mixtures of different milks, PDO products represent less than 1% of the eight thousand tons produced yearly in the country. As observed in the case of Spanish cheeses, these figures indicate that certified products have a relatively limited economic impact on the cheese market. Portuguese PDO cheeses were recently reviewed by Serrano et al. [98].

#### 2.2.1. Azeitão

*Azeitão* is a PDO ewe’s milk cheese produced in the foothills of the *Arrábida* mountains in southwestern Portugal. Its production is restricted to the municipalities of *Setúbal*, *Palmela*, and *Sesimbra*. Pinho et al. [99] investigated the evolution of FAAs and biogenic amines in *Azeitão* cheese stored at different storage temperatures over one month. The predominant FAAs identified were proline, valine, isoleucine, and leucine, while the main biogenic amines (BAs) identified were (mg/kg db): tyramine (122), cadaverine (181), histamine (458) and spermine (61.4). Storage at room temperature (25 °C) led to a significant increase in tyramine and putrescine levels. This highlights the necessity of maintaining a strict cold chain—from transport and distribution to retail and consumer storage—to minimise BA accumulation before consumption. Partidário et al. [85] evaluated the composition and nutritional quality of several PDO ewe’s milk cheeses and observed differences in fatty acid profiles, particularly in oleic acid content. Values of 15.4, 17.1, and 19.7 g/100 g of total FAs were reported for *Azeitão*, *Évora*, and *Nisa* cheeses, respectively. Differences were also observed in conjugated linoleic acid (CLA), with the total CLA reaching 1.15 g/100 g FA in *Nisa* cheese, whereas *Azeitão* exhibited lower levels (0.90 g/100 g FA). *Nisa* cheeses showed significantly higher contents of oleic and stearic acids, MUFAs, and CLA. Although the relatively high CLA content and the low ω6/ω3 ratio suggest favourable health-related properties, the polyunsaturated-to-saturated fatty acid ratio (PUFA/SFA) was consistently low (0.06), remaining below the recommended dietary guideline (0.45). Rocha [100] and Rocha et al. [101] analysed the diversity of LAB in *Azeitão* and *Nisa* cheeses produced in different cheesemaking plants. Colony-forming unit counts remained consistent throughout the three years of the study. *Lactococcus* spp. exhibited the highest bacterial counts, whereas *Enterococcus* spp. showed the lowest. RAPD-PCR analysis revealed no consistent trends in bacterial diversity. *Enterococcus* spp. displayed the greatest diversity despite having the lowest CFU counts. Identification of enterococcal isolates by PCR revealed a predominance of *E. faecium* (53.8%), followed by *E. faecalis* (25.5%) and *E. durans* (20.7%). The pathogenic potential of representative isolates was evaluated by assessing antibiotic resistance and virulence factors. Significant differences in resistance patterns were observed across production years for both lactococci and enterococci. A non-intrinsic resistance to clindamycin and erythromycin was identified among LAB isolates. Additionally, enterococci resistant to teicoplanin, ciprofloxacin, tetracycline, chloramphenicol, erythromycin, and vancomycin were identified. Although isolates resistant to three or more antimicrobials were identified, none met the criteria for classification as multidrug-resistant bacteria. Overall, no consistent patterns in bacterial counts or microbiome diversity were identified over time in the artisanal cheeses studied. Despite the detection of some concerning resistance traits among enterococci, no multidrug-resistant isolates were observed.

Recently, Serrano et al. [102] explored why *Azeitão* and *Nisa* cheeses maintain consistent regional characteristics despite the absence of commercial starter cultures. The study found that all bacterial isolates presented the ability to form biofilms. These biological structures allow bacteria to colonise and persist on surfaces within cheese factories. According to the authors, this persistence creates a “*microbial heritage*” unique to each production facility, effectively acting as a natural, built-in starter culture. The researchers established a direct link between the strength of biofilm formation and the long-term viability of these essential microbial cells.

Because these cheeses rely on the activity of wild bacteria, monitoring the safety of these strains, particularly enterococci, is a priority. Rocha et al. [103] conducted a genomic and phenotypic analysis of 145 enterococci isolates (*E. faecium*, *E. faecalis*, and *E. durans*) from *Azeitão* and *Nisa* cheeses. High levels of resistance were noted regarding tetracycline, teicoplanin, and quinupristin–dalfopristin. Critically, some resistance was found against clinically relevant classes, including β-lactams and aminoglycosides. Two isolates were classified as multidrug-resistant. So, while these traditional foods are considered safe due to a lack of reported infections linked to PDO cheese consumption, the researchers emphasised the need for surveillance of antibiotic microbial resistance. The primary concern is the potential for horizontal gene transfer, where antibiotic resistance genes could move from cheese bacteria to human pathogens along the food chain.

In the case of *Azeitão* cheese, the predominant bacteria identified by metagenomic analysis were *Lactococcus* spp., *Leuconostoc* spp., and *Lactobacillus* spp.

#### 2.2.2. Beira Baixa

*Beira Baixa* cheeses comprise: *Amarelo da Beira Baixa*, *Castelo Branco*, and *Picante da Beira Baixa*.

*Amarelo da Beira Baixa* is a semi-hard PDO cheese with a minimum ripening period of 45 days. It is produced from a mixture of ewe’s milk (50–100%) and goat’s milk (0–50%) and coagulated using either animal or vegetable rennet. Gomes [104] studied the evolution of the physicochemical characteristics of *Amarelo da Beira Baixa* and *Picante da Beira Baixa* cheeses produced by a PDO-recognised dairy. *Amarelo da Beira Baixa* cheese samples were analysed at days 5, 30, and 45 of ripening, whereas *Picante* cheese was evaluated at days 45, 90, and 120. The results revealed clear physicochemical differences between the two cheese types. Based on these findings and previously published data, the author suggested that recent changes in production technologies may significantly influence the physicochemical properties of these traditional cheeses.

Rodrigues [105] investigated the microbiological quality of *Amarelo da Beira Baixa* PDO cheese and the effect of production season. Twenty cheese samples were analysed. All cheese samples were negative for *Salmonella* spp. and *L. monocytogenes*, which indicates compliance with key food safety standards. Psychrotrophic microorganisms were the dominant group in all cheeses, followed by LAB. Yeasts consistently outnumbered moulds, with counts significantly higher in winter compared to spring. For most microbial groups, including LAB, Enterobacteriaceae, coliforms, and psychrotrophic bacteria, mean counts were higher in winter than in other seasons, although these differences were generally not statistically significant. Overall, the results highlight the predominance of psychrotrophic microorganisms and yeasts in *Amarelo da Beira Baixa* cheese and suggest a moderate influence of seasonal variation on microbial composition during ripening.

In recent years, *Queijos da Beira Baixa* have been extensively studied to define nutritional standards and microbial fingerprints, which has reinforced their standing as a high-quality regional product. Santos et al. [106] conducted a comparative analysis between certified *Amarelo da Beira Baixa* PDO cheeses and their non-certified counterparts. The PDO cheeses revealed a total lipid content of ca. 32%, protein levels of 21%, and an energetic value of 368.3 kcal/100 g. In terms of fatty acid composition, saturated fats (SFAs) averaged 18.6%, while MUFAs and PUFAs were 8.8% and 1.4%, respectively. Interestingly, the study found that only specific parameters—moisture-to-dry-extract ratio, protein, pH, and two fatty acids (C18:2 and C20:0)—showed statistically significant differences compared to the non-certified cheeses. These findings underscore a consistent and high-standard production process in the region, suggesting that more local producers could successfully meet the criteria for PDO certification.

The biological complexity of *Amarelo da Beira Baixa* cheeses was further elucidated by Cardinali et al. [107] through metataxonomic and physicochemical analysis. The study revealed a diverse microbial ecosystem. Regarding bacterial communities, *L. lactis* was the dominant species across all samples, supported by other taxa such as *Lactiplantibacillus plantarum*, *Streptococcus thermophilus*, and *Lacticaseibacillus zeae*. LAB count reached concentrations up to 9 log CFU/g. The fungal population was dominated by *Candida sake*, *D. hansenii*, and *Pichia kluyveri*. Regarding the chemical profile, pH values ranged between 4.72 and 5.85, with carboxylic acids and esters considered the primary volatile organic compounds (VOCs) responsible for the cheese’s aromatic complexity. Physically, the cheeses exhibited significant structural integrity, with hardness values ranging from 38.3 to 68.55 N. Together, these studies highlight that the unique sensory and nutritional properties of *Amarelo da Beira Baixa* cheeses are a direct result of their specific microbial dominance and well-established artisanal practices.

More recently, Serrano et al. [98] reported that the predominant LAB in *Amarelo da Beira Baixa* are *L. lactis* and *Lactiplantibacillus plantarum*. Other bacteria include *Lacticaseibacillus zeae*, *Streptococcus thermophilus*, and *Loigolactobacillus coryniformis*. Regarding fungi, *Candida sakey*, *Ustilago, Starmerella*, *Cladosposium variabile*, and *Pichia kluyveri* were reported.

*Castelo Branco* cheese is made with raw ewe’s milk coagulated with *C. cardunculus* and ripened for at least 40 days. Traditionally, milk from pure-breed *Merino da Beira Baixa* was used, but exotic breeds such as *Assaf* and crossbreeds of *Merino* and *Assaf* breeds are also employed. Ferreira et al. [84] compared the volatile profile and sensory characteristics of winter-produced *Castelo Branco* cheeses made from *Merino*, *Assaf*, and crossbreed milk. The volatile profiles differed significantly among the three types, although the microbiological characteristics were generally similar. *Merino* milk cheeses contained all acids in higher relative amounts than *Assaf* or crossbreed cheeses, which contributes to the characteristic ewe aroma of *Castelo Branco* cheese. *Assaf* cheeses contained fewer carbonyl compounds than *Merino* and crossbreed milk cheeses, which had similar profiles for several ketones. The relative proportions of alcohols varied, with *Assaf* milk cheeses containing the highest levels. Esters, which are typically floral and fruity, help modulate the sharpness and bitterness contributed by fatty acids and amines. The authors noted that lower-quality ewe’s milk cheeses tend to have higher levels of sulphur compounds and 3-methylbutanal, whereas higher-quality cheeses have more esters and free fatty acids. Sensory analysis revealed significant differences between *Merino* and both *Assaf* and crossbreed milk cheeses, while no significant differences were observed between *Assaf* and crossbreed products.

*Picante da Beira Baixa* is a hard, spicy and salty cheese, with a minimum ripening period of 120 days, traditionally manufactured at the farm level in Portugal. The cheese can be made using variable proportions of ewe’s and goat’s milk and different types of milk-clotting agents.

Freitas et al. [108] compared the influence of milk composition, coagulant type, salt content, and ripening time on the microbiological and physicochemical characteristics of *Picante da Beira Baixa* cheeses. Two milk mixtures (80:20 and 60:40 ewe: goat), two levels of salt, two coagulants (animal and vegetable rennet), and two ripening periods (30 and 120 days) were compared. Milk type significantly influenced the counts of viable microorganisms and the extent of proteolysis, likely due to the higher initial microbial load in goat’s milk. The type of coagulant had a pronounced effect on proteolysis: cheeses coagulated with vegetable rennet exhibited a greater breakdown of αS1-casein and β-casein and higher water-soluble nitrogen values than those coagulated with animal rennet, while differences in NPN levels were smaller.

**Figure 3:**
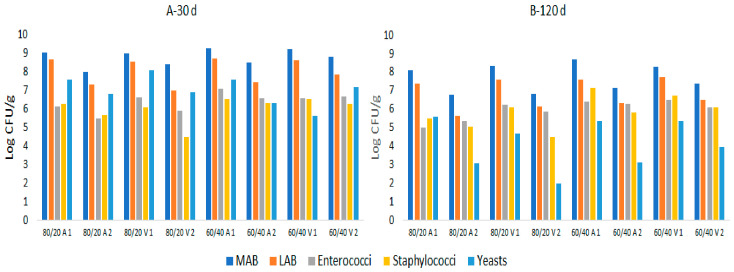
Average counts of microbial groups of *Picante da Beira Baixa* cheeses produced with different manufacturing parameters after 30 (**A**) and 120 (**B**) days of ripening; 80/20 and 60/40 ewe: goat milk proportions; A: animal rennet; V: vegetable rennet; 1 and 2: number of salt additions (based on data from Freitas et al. [111]).

Salt content was a statistically significant factor affecting all measured microbiological and physicochemical characteristics of *Picante da Beira Baixa* cheese. Counts of mesophilic aerobic bacteria, LAB, and yeasts were significantly influenced by milk composition, salt content, and ripening time, while viable staphylococci were affected by milk type, coagulant, and salt content, but not by ripening. Enterobacteriaceae were identified at 30 days in cheeses with lower salt and a lower pH but were absent by 120 days. Vegetable rennet tended to favour enterococci growth, whereas animal rennet promoted staphylococci proliferation. The evolution of LAB and yeasts during ripening was strongly influenced by salt content, with vegetable rennet leading to higher yeast counts, particularly in cheeses containing 20% goat milk (Figure 3).

The evolution of the physicochemical and biochemical properties of *Picante da Beira Baixa* cheese throughout ripening was further investigated by Freitas et al. [109,110]. WSN and NPN increased significantly during ripening, with WSN reaching 25% and 29% of the total nitrogen by 180 days in plain ewe’s and goat’s cheeses, respectively. The maximum NPN values were attained at 180 days, corresponding to 87% and 92% of the WSN. FAAs were dominated by valine, leucine, and phenylalanine, which collectively accounted for 36–57% of the total FAAs, depending on milk composition and ripening time. SCFAs (C4–C8) contributed to the characteristic piquant flavour, while LCFAs (C10, C16, C18, and C18:1) were also present throughout ripening [109]. The total FAA concentration increased with ripening time, regardless of milk composition, coagulant type, or salting regime, which indicates that ripening time exerts the greatest influence on proteolysis [111,112]. The dominant FAAs in *Picante* cheese were valine, leucine, and phenylalanine, each representing more than 10% of the total FAA pool. Total FAAs increased steadily over time, regardless of milk composition, coagulant type, or salting, which indicates that ripening time exerts the strongest influence on proteolysis. Proteolysis was the most intense between 3 and 6 months. Cheeses made with 20% goat milk, coagulated with animal rennet and salted once, exhibited the highest total FAA content at 120 days. Higher proportions of ewe’s milk generally enhanced total FAAs, particularly by 120 days, while vegetable rennet produced slightly higher FAAs at 30 days, especially in cheeses with lower salt content. By 120 days of ripening, cheeses coagulated with animal rennet showed greater FAA levels than those produced with vegetable rennet, which confirms that vegetable rennet contributes less to FAA release. According to Freitas and Malcata [111], proteolysis in *Picante* cheese progresses gradually in cheeses made with animal rennet, whereas cheeses made with vegetable rennet showed no significant changes in WSN between 30 and 120 days. Cheeses coagulated with vegetable rennet exhibited higher WSN than those with animal rennet but lower total and protein-adjusted nitrogen (TASN and PTASN). Overall, ripening time had the strongest effect on FAA accumulation compared to milk composition, coagulant type, or salting, and vegetable rennet contributed less to FAA release than animal rennet.

The same authors reported that LAB and yeasts dominated throughout ripening, with viable counts above 7 log CFU/g. The main LAB species were enterococci (*E. faecium*, *E. faecalis*, and *E. durans*) and lactobacilli (*L. plantarum* and *L. paracasei*), while yeasts were primarily *D. hansenii* and *Y. lipolytica*. Metabolic studies of four bacterial species (*E. faecium*, *E. faecalis*, *L. plantarum*, and *L. paracasei*) and three yeasts (*D. hansenii*, *Y. lipolytica*, and *Cryptococcus laurentii*) previously isolated from *Picante* cheese showed that lactic acid production, correlated with lactose degradation, was highest for *L. paracasei*, followed by *E. faecium*. Citrate metabolism was observed in *E. faecalis* and, to a lesser extent, in *E. faecium*, *L. plantarum*, and *L. paracasei*, while *Enterococcus* and *L. plantarum* produced relatively high amounts of formic acid. A mixed starter composed of *E. faecium*, *L. plantarum*, and *D. hansenii* exhibited synergistic effects compared to individual strains. Among LAB, *L. paracasei* was the dominant lactic acid producer and was considered the most suitable strain for a starter tailored to *Picante* cheese. The presence of citrate-metabolising strains throughout ripening highlighted their critical role in glycolysis and flavour development [111].

#### 2.2.3. Évora

*Évora* is a PDO ewe’s milk cheese produced with raw milk. It is typically manufactured in small, flat cylindrical formats weighing 60–90 g, although larger sizes (120–300 g), known as *Merendeira*, share the same shape but have a larger diameter.

Potes [113] evaluated the microbiological evolution of *Évora* cheese during ripening across two production seasons. LAB reached higher counts earlier in spring than in winter, whereas the maximum LAB levels in winter were only achieved at the end of ripening. Enterobacteria and coliform counts also showed seasonal differences, decreasing more rapidly during spring. LAB represented approximately 95% of the microbiota (mainly *Lactobacillus* spp., *Lactococcus* spp., and *Enterococcus* spp.). *E. faecium* was the predominant species, followed by *E. faecalis* and the *E. hirae*/*durans* complex. *Lactobacillus* spp. became more prevalent at later ripening stages, while *Lactococcus* spp. declined. Yeasts, mainly with lipolytic activity, were suggested to play an important role in cheese maturation, which is characterised by intense lipolysis. *Listeria monocytogenes* and *Salmonella* spp. were not identified.

Pereira-Dias et al. [114] investigated yeast evolution during the ripening of *Évora* cheese, reporting counts between 2.7 and 6.4 log CFU/g, with maximum values observed at 30 days of ripening. Esterase activity was widespread among isolates, whereas proteolytic activity was identified in only a small proportion of strains. Yeast diversity decreased over ripening, with *D. hansenii* and *Candida intermedia* becoming dominant in mature cheeses.

Torres [83] analysed the volatile composition of *Évora* cheese at three maturation stages (curd, 21, and 42 days) and identified 20 compounds. Free fatty acids were the main volatiles, which indicates extensive lipolysis. Butanoic and hexanoic acids predominated, contributing to pungent and rancid odours, while acetic acid was also abundant. During ripening, the concentrations of acetic, butanoic, hexanoic, octanoic, 2,4-hexadienoic, and decanoic acids increased. Branched-chain fatty acids, such as isovaleric acid, rose mainly during the first 21 days, contributing to rancid, buttery, and sweet notes. Esters influenced aroma even at low concentrations: ethyl hexanoate increased throughout ripening, whereas ethyl decanoate decreased at 21 days and then stabilised. Alcohols included 2,3-butanediol isomers, from lactose and citrate metabolism, and methylated alcohols derived from branched-chain FAAs. In addition, 3-methylbutanol, from leucine, peaked at 21 days, imparting sweet, fresh notes, and 2-phenylethanol, from phenylalanine, contributed to floral aromas and was linked to yeast and lactococcal activity. Aliphatic aldehydes, formed from fatty acid autoxidation, were quickly converted to alcohols or acids, with octanal identified only at 42 days. Acetoin, from citrate and lactose metabolism, increased throughout ripening, contributing to buttery notes.

Carvalho et al. [115] indicate that longer ripening resulted in a lower pH, moisture content, and a_w_ and higher salt-in-moisture values. *Évora* cheeses ripened for 120 days exhibited a more intense aroma, spiciness, firmness, and granular texture, although overall consumer acceptance remained high across all ripening stages. These authors also investigated how maturation time (30, 60, and 120 days) under controlled conditions (14–15 °C; 65–70% humidity) affects the chemical and sensory profile of *Évora* cheese. The 120-day stage received the highest scores for crust colour, aroma pungency, taste intensity (piquancy), and granular texture. Interestingly, while panellists clearly distinguished between the stages, with higher agreement on the characteristics of older cheeses, the overall acceptance remained positive across all ripening periods. This suggests that the degree of ripeness shifts the cheese’s character but does not diminish its appeal to informed consumers.

Machado [116] assessed the impact of three *C. cardunculus* ecotypes and animal rennet on the sensory characteristics of *Évora* cheese using descriptive and hedonic tests with a trained panel. Significant differences were observed in 45-day-old cheeses for crust uniformity, upper and lateral bulging, and overall appearance. At 60 days, differences persisted in upper and lateral bulging, and at 90 days, differences persisted in upper and lateral bulging as well as in overall appearance. Hedonic tests further revealed that the appearance, aroma, and texture varied with the type of coagulating agent, with cheeses made using animal rennet clearly distinguishable from those produced with vegetable rennet by both panels.

According to Pinheiro et al. [117], the choice of coagulant is a defining factor in the biochemical identity of *Évora* cheese. The authors compared the proteolytic effects of three local cardoon ecotypes and of a commercial animal rennet over 60 days. Cheeses made with vegetable coagulants (*C. cardunculus*) showed significantly higher protein degradation than those made with animal rennet. Proteolysis accelerated during the first 35 days of ripening and stabilised afterwards. By the end of the 60-day study, αS_1_-caseins were more heavily degraded (54.90%) compared to β-caseins (37.27%). This specific breakdown pattern, facilitated by the cardoon’s enzymes, was considered responsible for the unique texture and flavour development that distinguishes traditional *Évora* cheese from those made with industrial rennet.

#### 2.2.4. Nisa

The *Nisa* PDO cheese is made with raw ewe’s milk from the *Merina Branca* ewe breed, coagulated with *C. cardunculus*. It is a cheese with a semi-hard paste and with a yellowish-white colour. Ripening occurs in two stages: the first lasts 15–20 days at 8–10 °C and 80–90% relative humidity, and the second lasts 30–40 days at 10–14 °C and 85–90% relative humidity. The cheese is produced in two formats: *Merendeira*, 10–12 cm in diameter and 200–400 g, and the original format, 13–16 cm in diameter and 800–1300g. Production is restricted to an area in Northern *Alentejo* that encompasses eight municipalities [118].

Cardinali et al. [119] aimed to identify the natural bacterial populations within *Nisa* PDO samples using viable counts and metataxonomic profiling. The research also evaluated the cheese’s physicochemical and morpho-textural properties, alongside analysing volatile organic compounds via solid-phase microextraction combined with gas chromatography–mass spectrometry (SPME-GC-MS). The results showed pH levels ranging from 4.84 to 5.74, lactic acid concentrations between 0.83 and 2.10 g/100 g, and a_w_ ranging from 0.942 to 0.960. The hardness of the cheeses varied from 34.45 N to 126.05 N. Microbiological testing revealed lactococci and lactobacilli populations reaching 9.01 log CFU/g, while coagulase-negative cocci and enterococci peaked at 7 log CFU/g. Sequencing indicated a high prevalence of *Lactococcus lactis* and *Leuconostoc mesenteroides* across all samples, along with *Lactiplantibacillus plantarum*, *Lactococcus piscium*, and *Lacticaseibacillus zeae*. The primary VOCs identified were carboxylic acids, carbonyls, alcohols, and esters, which demonstrated significant correlations with the bacterial communities.

#### 2.2.5. Serra da Estrela

*Serra da Estrela* cheese is the most renowned Portuguese PDO cheese made from raw ewe’s milk, obtained exclusively from the *Bordaleira Serra da Estrela* and *Churra Mondegueira* breeds. Milk coagulation is achieved using *C. cardunculus*, and cheeses are ripened for at least 30 days. It is the most extensively studied Portuguese PDO cheese. *Serra da Estrela Velho*, with a minimum ripening time of 90 days, was also classified as a PDO cheese.

**Figure 4:**
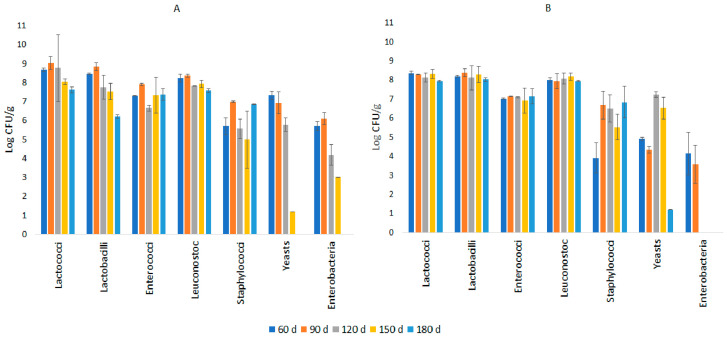
Microbial groups of *Serra da Estrela* cheese made with non-refrigerated milk (**A**) and refrigerated milk (**B**) over 180 ripening days (based on data from Dahl, Tavaria and Malcata [69]).

The diversity of microbiological and chemical profiles typical of raw milk cheeses was confirmed for *Serra da Estrela* cheese by Dahl, Tavaria and Malcata [69]. Figure 4 summarises the viable counts of lactococci, lactobacilli, leuconostoc, enterococci, yeasts, Enterobacteriaceae, and staphylococci in *Serra da Estrela* cheeses produced from non-refrigerated (Figure 4A) and refrigerated milk (Figure 4B).

Lactic acid bacteria dominated the microbiota throughout ripening. Cheeses produced from refrigerated milk showed significantly lower counts of Enterobacteriaceae and enterococci than those produced from non-refrigerated milk, which indicates that milk refrigeration could limit the growth of undesirable microorganisms. However, refrigeration may also promote the growth of psychrotrophic bacteria, such as *Pseudomonas* spp., potentially contributing to cheese defects through excessive proteolysis. According to these authors, the volatile profile of *Serra da Estrela* cheese included short-chain carboxylic acids such as acetic, propionic, isobutyric, and isovaleric acids, which are associated with amino acid catabolism via oxidative deamination. Semi-volatile fatty acids and their corresponding ethyl esters were also identified, likely resulting from the lipolytic activity of yeasts and Enterobacteriaceae. Ethyl esters, which impart fruity aromas, were particularly pronounced in cheeses made from refrigerated milk. Elevated levels of ethyl octanoate and ethyl decanoate indicated yeast-driven lipolysis rather than amino acid degradation. All cheeses exhibited a progressive increase in volatile SCFAs during ripening. Additionally, compounds such as 1-octanol and 2-nonanone, associated with LAB metabolism, were identified after 150 days of ripening, coinciding with LAB dominance.

Further insights into the microbiology of *Serra da Estrela* cheese were provided by Tavaria and Malcata [120], who reported significant effects of geographical location on the viable counts of lactococci, lactobacilli, enterococci, Enterobacteriaceae, and staphylococci. By contrast, the year of manufacture significantly influenced only yeast and staphylococcal counts. Enterobacteriaceae, typically associated with poor hygienic conditions, decreased during ripening, which suggests effective control by competing microbial groups that prevail at later ripening stages. Enterococci count, not affected by the year of manufacture, was strongly influenced by geographical location and ripening time. Their numbers increased from approximately 7 log CFU/g at day 1 to 8 log CFU/g at day 18, followed by a decrease to around 7 log CFU/g by day 60, remaining high throughout ripening and following trends like those of lactococci. Lactobacilli increased from about 7 log CFU/g on day 1 to 8 log CFU/g on day 35, decreasing slightly thereafter but never falling below 8 log CFU/g. Both geographical location and ripening time significantly affected lactobacilli viability. Lactococci exhibited a comparable pattern, increasing from ca. 8 log CFU/g on day 1 to 9 log CFU/g on day 35 and day 60. Staphylococcal counts averaged ca. 6 log CFU/g in the first year of the study. In the second year, counts decreased to approximately 4 log CFU/g by 35 days and increased again to around 5 log CFU/g at the end of ripening. These levels were not considered a health risk, as only 14% of the isolates were identified as *S. aureus*. Moreover, the authors explained that staphylococcal enterotoxins require viable cell counts above 7 log CFU/g to be identified and could be inactivated during prolonged ripening. Yeast counts were not significantly affected by either geographical location or year of manufacture and were relatively uniform across the PDO region. This homogeneity was attributed to their predominant airborne origin. Yeasts play a relevant role in *Serra da Estrela* cheese by metabolising lactic acid and enhancing proteolysis and lipolysis, thereby contributing to flavour development [69]. Tavaria et al. [16] evaluated amino acid and soluble nitrogen evolution throughout the ripening of *Serra da Estrela* cheese.

Tavaria, Reis and Malcata [15] also evaluated *Serra da Estrela* cheeses ripened under control conditions at 60, 90, 120, 150, and 180 days. *Lactococcus* was the dominant genus, with viable counts above 8 log CFU/g at up to 120 days of ripening. Milk refrigeration was evaluated as a strategy to limit microbial proliferation between milking and cheesemaking; however, as previously noted, this practice may promote the growth of psychrotrophic bacteria capable of producing highly active proteolytic and lipolytic enzymes. Both farmhouse of origin and ripening time were statistically significant factors influencing bacterial viability, regardless of microbial group. LAB dominated throughout ripening across all dairies, with counts ranging from 7 to 9 log CFU/g and generally increasing with ripening time. Milk refrigeration significantly affected Enterobacteriaceae and lactobacilli populations: cheeses made from non-refrigerated milk showed higher Enterobacteriaceae counts, whereas lactobacilli counts were lower. Milk refrigeration appears to promote yeast growth. These yeasts are thought to play a key role in utilising lactic acid and accelerating the breakdown of proteins and triglycerides, ultimately contributing to the development of specific flavours. Other microbial groups, predominantly LAB, exhibited higher viable numbers in cheeses produced from non-refrigerated milk at up to 90 days of ripening; thereafter, higher counts were observed in cheeses made from refrigerated milk. Microbial viability in *Serra da Estrela* cheese was strongly dependent on the farmhouse of manufacture. Milk refrigeration contributed to the control of Enterobacteriaceae but was also associated with differences in the cheese microstructure. Although no major microstructural changes were identified during ripening, cheeses produced from refrigerated milk exhibited a loose protein matrix, in which lactobacilli and yeasts predominated.

**Figure 5:**
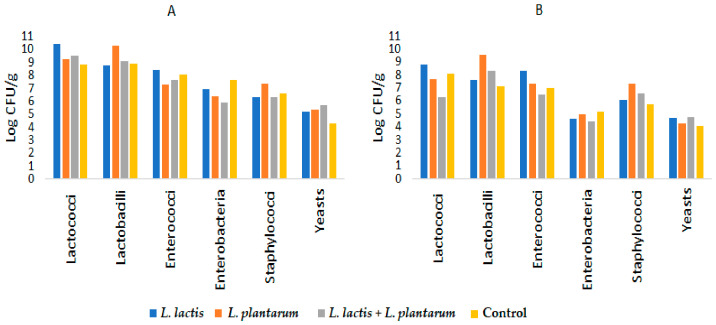
Average counts (log CFU/g) of the main microbial groups of *Serra da Estrela* cheese made with different starter cultures after 28 ripening days (**A**) and after 63 ripening days (**B**) (based on data of Macedo, Tavares and Malcata [121]—(Ll) cheese added with 1% *Lactococcus lactis*; (Lp) cheese added with 1% *Lactobacillus plantarum*; (Ll + Lp) cheese added with 0.5% *Lactobacillus plantarum* and 0.5% *Lactococcus lactis*).

Macedo, Tavares and Malcata [121] produced experimental batches of *Serra da Estrela* cheeses from raw ewe’s milk, inoculating milk with wild strains of *L. lactis* ssp. *lactis* and *Lactobacillus plantarum* either individually or in combination. These strains were previously isolated from the native microbiota of *Serra da Estrela* cheese. A control batch was produced in parallel, without starter addition. The evolution of the viable counts of the main microbial groups is summarised in Figure 5. The sensory attributes of the ripened cheeses were also evaluated. Cheeses produced with starter cultures showed significantly lower viable counts of Enterobacteriaceae than control cheeses. By contrast, the addition of *L. lactis* or *L. plantarum* led to significant increases in enterococci and staphylococci counts. Proteolysis, assessed through WSN and PTASN, was not significantly affected by starter addition relative to the control, although *L. lactis* significantly increased TCASN. Control cheeses received slightly higher scores for flavour and texture than cheeses produced with starters. The presence of *L. lactis* promoted the growth of enterococci to a greater extent than *L. plantarum*.

The authors concluded that the combined use of *L. lactis* and *L. plantarum* was the most effective strategy for controlling Enterobacteriaceae growth. However, starter addition did not reduce staphylococci counts as expected.

Tavaria et al. [21] also evaluated four batches of *Serra da Estrela* cheese from different dairies to assess the evolution of free amino acids (FAAs) and other proteolysis indicators. Water-soluble nitrogen increased steadily, reaching up to 43% of the total nitrogen by 180 days of ripening, reflecting the intense proteolytic activity associated with the vegetable coagulant (*C. cardunculus*). Total casein-soluble nitrogen (TCASN) ranged from 16 to 20% of TN, which supports the view that cardoon markedly enhances the pool of fermentable nitrogenous substrates in this cheese. Protein-adjusted soluble nitrogen (PTASN), an indicator of FAA concentration, also increased during ripening, reaching approximately 12% of TN, with the most pronounced rise occurring between 60 and 90 days of ripening. By 180 days of ripening, glutamic acid, valine, leucine, and lysine were the predominant FAAs, accounting together for 56–70% of the total FAAs across all dairies. Cheeses produced with refrigerated milk exhibited higher levels of γ-aminobutyric acid (GABA) and lower levels of glutamic acid compared to those made from non-refrigerated milk. Both dairy farm and ripening time were statistically significant factors affecting all nitrogen fractions, explaining much of the compositional heterogeneity observed among cheeses. Milk refrigeration also significantly influenced most proteolytic indices, except for TCASN.

Table 5 presents the significative correlations found between compounds with a potential impact on cheese flavour and the main microbial groups found in *Serra da Estrela* cheese identified in the research conducted by Tavaria et al. [21].

Viable LAB in *Serra da Estrela* cheese remain high throughout ripening (8–9 log CFU/g up to 180 days), with higher counts in cheeses produced from refrigerated milk [69]. The fastest increase in free amino acids (FAAs) occurred early after manufacturing (days 3–7), while the greatest overall accumulation was observed between days 18 and 35, coinciding with exponential LAB growth, which supports their role in flavour development. Cheeses produced from refrigerated and non-refrigerated milk showed similar FAA profiles between 60 and 180 days of ripening, which differed quantitatively. Nitrogen fraction analysis indicates extensive but shallow-to-moderate proteolysis characterised by the preferential formation of polypeptides and large peptides, reflected in high WSN and TCASN. The greatest increases in these fractions occurred within the first three days after manufacturing, whereas FAA accumulation (PTASN) peaked between 7 and 18 days, which further highlights the contribution of LAB to flavour formation through amino acid catabolism.

Sousa and Malcata [122] compared three versions of *Serra da Estrela*-style ovine cheese made using raw, pasteurised, and pasteurised with starter culture addition. While raw milk yielded higher microbial counts, physicochemical properties remained similar across all types. Pasteurisation did not significantly impact protein breakdown initially; however, after 28 days, PS cheeses showed higher TCA and PTA nitrogen fractions and distinct electrophoretic profiles compared to the other two groups.

Other studies have demonstrated that the FAA profiles of *Serra da Estrela* cheese can be effectively used to discriminate producers, geographical origin, and production periods [123]. Across all samples, 17 FAAs were quantified, including eight essential amino acids (histidine, leucine/isoleucine, lysine, methionine, phenylalanine, threonine, tryptophan, and valine) and nine non-essential amino acids (arginine, asparagine, aspartic acid, cysteine, glutamic acid, glutamine, proline, serine, and tyrosine). Absolute FAA concentrations, essential FAA content, branched-chain amino acids (leucine, isoleucine, and valine), and FAA ratios (mg/g protein) were successfully applied as discriminant variables for producer identification.

Reis and Malcata [124] found that proteolysis in *Serra da Estrela* cheese was negatively correlated with microstructural parameters. Light microscopy showed matrix rearrangements without differences on pore numbers at up to 21 days, while electron microscopy revealed a reduction in larger pores (>40 μm^2^), explaining the decrease in overall porosity.

Partidário et al. [85] investigated the evolution of FFAs, triglycerides, and volatile compounds in *Serra da Estrela* cheese during ripening. FFAs increased mainly during the first three weeks of ripening, although overall lipolytic activity remained moderate, which resulted in total FFA levels lower than those reported for other ewe’s milk cheeses. The ratio of volatile fatty acids to medium- and long-chain fatty acids highlighted the increasing relevance of the volatile fraction as ripening progressed. Analysis of the volatile compounds identified nine ketones (including acetoin and diacetyl), five organic acids, five alcohols, and one aldehyde. High concentrations of diacetyl and acetaldehyde, produced by indigenous LAB, were identified and were considered major contributors to the characteristic creamy and buttery flavour of *Serra da Estrela* cheese. Ketones with four to seven carbon atoms were also present. The total ketone concentration increased between the 21st and the 42nd days of ripening, concomitant with the decrease in total FFAs and SCFAs, which suggests fatty acid oxidation as their primary origin. Given the fatty acid composition of ewe’s milk and the relatively short ripening period of *Serra da Estrela* cheese (30–45 days), the lipid fraction and its primary degradation products, particularly volatile fatty acids, play a key role in the aroma of this cheese. Twenty FFAs were associated with desirable buttery notes rather than off-flavours. Volatile compounds derived from lactose and citrate metabolism, notably diacetyl and acetoin, were abundant in early ripening stages; acetoin levels decreased towards the end of ripening, likely due to its reduction to 2,3-butanediol. Acetic acid, originating mainly from citrate and sugar metabolism and, to a lesser extent, amino acid deamination, was also identified at high concentrations. In addition, 3-methylbutanol, derived from leucine catabolism, was identified in cheeses ripened for 21 and 42 days.

In recent years, scientific interest has shifted towards characterising the chemical and biological “fingerprints” of *Serra da Estrela* cheese to ensure its authenticity, safety, and nutritional quality. Reis Lima et al. [123] established that while the cheese is characterised by a high abundance of saturated fatty acids (67–76%), it also contains significant levels of monounsaturated (17–25%) and polyunsaturated (5–7%) fatty acids. Crucially, these fatty acid profiles, comprising 23 identified saturated and unsaturated acids, act as a chemical signature. By utilising linear discriminant models and simulated annealing algorithms, researchers could classify *Serra da Estrela* cheeses based on their manufacturing plant, geographical origin, and production date with predictive sensitivities between 71 and 88%. Such chemometric techniques are essential for protecting the “genuineness” of the product against fraud.

However, the final quality of *Serra da Estrela* cheese is not static; it is highly susceptible to seasonal and technical variations. Fogeiro et al. [125] demonstrated that the flock lactation stage is the primary driver of the cheese’s physicochemical properties, with early-stage lactation yielding protein-rich, low-fat products. While the type of cardoon flower used influences the fat content and rind colour, the seasonal shift in milk composition remains the dominant factor in determining the cheese’s final attributes.

The complexity of *Serra da Estrela* cheese is further enhanced by its diverse microbiota. Rampanti et al. [126] have mapped the *Serra da Estrela* cheese microbiome, identifying over 500 taxa. The bacterial population is dominated by LAB, specifically, *L. lactis*, *Lactococcus piscium*, and *Leuconostoc* spp., which often exceed concentrations of 8.8 log CFU/g. Specifically, 30 taxa were present in all analysed cheese samples, including species of *Leuconostoc* spp. and *Lactococcus* spp. for bacteria and *Candida* spp., *Debaryomyces* spp., and *Yarrowia* spp. for fungi. These taxa were cumulatively the most prevalent genera in *Serra da Estrela* PDO cheese (average relative abundance >10%). Ultimately, this characterisation study contributed to a better understanding of the microbial dynamics of this traditional PDO product—namely, the influence of raw materials on the cheese microbiome—and could assist producers interested in preserving the identity, quality, and safety of *Serra da Estrela* PDO cheese. Rocha et al. [127] reported that, in all samples analysed, lactococci, lactobacilli and *Leuconostoc* spp. predominated over enterococci strains. Moreover, lactococci and lactobacilli abundance increased alongside the production season, while enterococci dropped considerably in late manufacturing. Lastly, *Leuconostoc* spp. content remained unchanged in all analysed periods. A correspondence analysis showed that *L. paracasei*, *L. lactis*, *E. durans*, *E. faecium*, and *L. mesenteroides* are transversal in *Serra da Estrela* cheese manufacturing and were closely associated with milk, curd, and cheese matrices. Additionally, *L. casei*, *L. plantarum* and *L. curvatus* were specifically associated with cheese matrices, possibly active during ripening and contributing to the development of these cheeses’ organoleptic characteristics. These microorganisms, along with fungal populations dominated by *D. hansenii*, drive the enzymatic activities, such as esterase and aminopeptidase, responsible for the development of volatile organic compounds. Notably, species like *L. casei* and *L. plantarum* appear to be specifically associated with the ripening phase, contributing to the development of the cheese’s hallmark sensory characteristics.

The review by Serrano et al. [98] indicates that the predominant bacteria in *Serra da Estrela* cheese were reported as *Leuconostoc* spp., *Lactococcus* spp., *Lactobacillus* spp. and *Enterococcus durans*. Fungi included *Candida* spp., *Debaryomyces* spp., *Yarrowia* spp., *Starmerella*, *Vishniacozyma victoriae*, *Kurtzmaniella zeylanoide*, *Cladosporium variabile*, *Cutaneotrichosporon curvatus*, and *Metschnikowia fructicola*.

The presence of potential pathogenic microorganisms was evaluated in *Serra da Estrela* cheeses produced by three different producers. The species identified included *E. faecium*/*faecalis*, *Staphylococcus aureus*/*haemolyticus*, *Staphylococcus vitulinus*, *Staphylococcus lentus*, and *Aerococcus viridans*. *E. faecalis* and *E. faecium* are generally regarded as non-pathogenic members of the gastrointestinal microbiota of humans and animals [128] and are also classified as LAB [129]. Nevertheless, they may behave as opportunistic pathogens, being associated with nosocomial infections in humans and with several animal diseases, including bovine mastitis, diarrhoea in livestock, and septicaemia in poultry [130]. The colonisation of dairy herds by *S. aureus* and the subsequent contamination of raw milk remain major concerns for both dairy production and public health [131,132], as raw milk cheeses may act as vehicles for this pathogen [133]. *Staphylococcus haemolyticus*, the second most frequently isolated coagulase-negative staphylococci, is a skin commensal increasingly recognised as an opportunistic pathogen [134]. *S. lentus* and *S. vitulinus*, both coagulase-negative species belonging to the *Staphylococcus sciuri* group, are commonly associated with animal skin and food of animal origin and are considered opportunistic pathogens [135,136,137,138,139]. *Aerococcus viridans*, a Gram-positive, microaerophilic lactic acid bacterium, is widely distributed in the environment and has been described as an opportunistic pathogen in both clinical and agricultural settings [140,141,142].

#### 2.2.6. Serpa

*Serpa* is a full-fat PDO ewe’s milk cheese with a semi-soft, creamy texture and a characteristic strong flavour, often described as slightly hot and spicy, typical of cheeses produced with cardoon. It presents a minimum maturation index (WSN/TN) of approximately 45% and a moisture content ranging from 61 to 69% [143]. *Serpa* cheese presents a profile of high value and artisanal complexity, though it faces distinct regulatory and consistency challenges. Its production is governed by strict PDO standards that currently prohibit pasteurisation and the use of commercial starter cultures.

**Figure 6:**
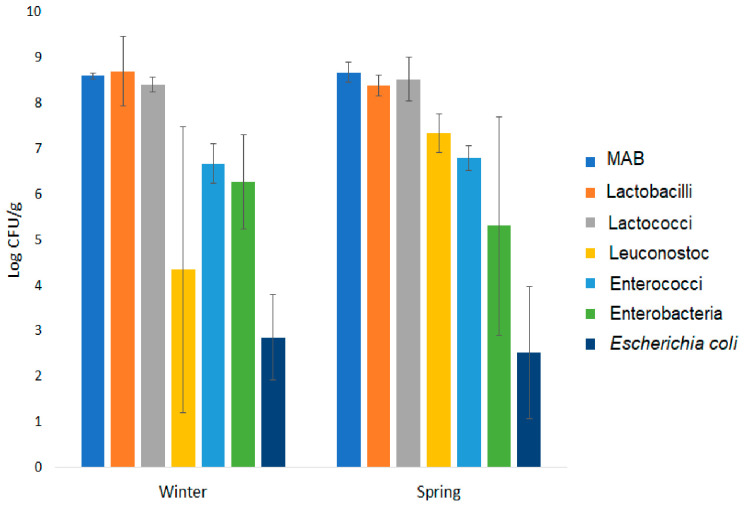
Bacterial groups of PDO *Serpa* cheese produced in winter and spring (based on data from Gonçalves et al. [62]). MAB: mesophilic aerobic bacteria. Results are expressed as means values ± standard deviation from 3 different cheese companies.

Gonçalves dos Santos et al. [62] investigated the microbiota of *Serpa* cheese using both culture-dependent and culture-independent (high-throughput sequencing, HTS) approaches. Culture-dependent analysis showed that LAB dominated the microbiota. *L. paracasei* and *Lactobacillus casei* were the predominant species in cheeses from PDO-registered producers, whereas *Lactobacillus brevis* was more prevalent in cheeses not certified as PDO (non-PDO). By contrast, HTS analysis revealed that *Lactococcus* was the most abundant genus (40–60%), followed by *Leuconostoc* and *Lactobacillus*. The results obtained for PDO *Serpa* cheeses are presented in Figure 6. Microbial counts were evaluated in spring and winter. Mesophilic aerobic bacteria ranged from 8.0 to 8.9 log CFU/g. Lactococci and lactobacilli were present at similar levels, whereas *Leuconostoc* spp. occurred at lower levels and showed the greatest variability among producers and seasons. Enterococci were identified at relatively high levels (6.30–7.65 log CFU/g). Among secondary microbiota, staphylococci were present at significantly lower levels in winter than in spring.

The microorganisms isolated on PCA medium consisted mainly of LAB and, to a lesser extent, enterobacteria. *L. paracasei* and *L. casei* predominated in PDO cheeses in both seasons, together with *L. plantarum*, *L. mesenteroides* (identified only in spring), and *Enterococcus* spp. Enterobacteriaceae accounted for approximately 20–40% of the total isolates, with *Hafnia alvei* representing 20–30% across all producers. Enterobacteria persisted until the end of ripening and contributed to sensory development. *E. coli* followed a similar trend but at much lower counts. At the end of ripening, lactococci were recovered in low numbers. Isolates recovered on MRS agar were mainly *Lactobacillus* spp., with *L. paracasei* and *L. casei* as the dominant species. Other species, including *L. plantarum*, *L. brevis*, *L. pentosus*, and *L. curvatus*, were sporadically identified. *L. mesenteroides* and enterococci (*E. faecalis*, *E. faecium*, and *E. hirae*) were also identified, but not consistently across samples. On M17 agar, only *L. lactis* was isolated, which contrasts with HTS results that identified *Lactococcus* as the dominant genus. Al *Leuconostoc* isolates were identified as *L. mesenteroides*, a species associated with aroma development through citrate and lactate metabolism [144]. The enterococcal population was dominated by *E. faecalis*, followed by *E. faecium*, while *E. hirae* was identified only in winter cheeses. *Staphylococcus epidermidis*, *S. warneri*, and *S. cohnii* were mainly isolated from spring cheeses. Staphylococci are commonly part of the secondary cheese microbiota and could persist throughout ripening due to their tolerance to salt and low a_w_ [65]. *Hafnia alvei*, frequently reported as the dominant Gram-negative bacterium in cheeses, exhibits strong proteolytic activity that may influence sensory attributes such as creaminess [65,66]. Importantly, major foodborne pathogens (*L. monocytogenes*, *Salmonella* spp., and enterohaemorrhagic *E. coli*) were not identified after 30 days of ripening.

The yeast community of *Serpa* cheese was also characterised by Gonçalves dos Santos et al. [145] using the same methodologies. Sixteen cheese batches from PDO and non-PDO producers were analysed in spring and winter. Yeast counts were approximately 5 log CFU/g in winter and generally lower in spring. At the end of ripening, the dominant yeasts were *D. hansenii* and *Kluyveromyces marxianus*, with *Candida* spp. and *Pichia* spp. identified at lower levels. HTS confirmed the predominance of *Debaryomyces* spp. and *Kluyveromyces* spp. but also revealed an important presence of *Galactomyces* spp., suggesting that it plays a role during early ripening stages. PDO cheeses were consistently dominated by *D. hansenii* and *Kluyveromyces* spp., whereas non-PDO cheeses showed a distinct yeast profile, including *Candida cabralensis* and *Moniliella suaveolens*. The recurrence of similar yeast communities across seasons in PDO cheeses suggests that the cheesemaking environment may act as a key source of the yeasts involved in ripening. Overall, both methodological approaches confirmed *Debaryomyces* spp. and *Kluyveromyces* spp. as the dominant genera, widely recognised as safe and beneficial for cheese ripening and sensory quality. Roseiro et al. [146] evaluated the proteolysis in traditionally and semi-industrially produced *Serpa* cheeses. Proteolysis was shown to depend on cheesemaking practices, particularly coagulant activity, salting, and ripening conditions. The degradation of αS_1_- and β-caseins was consistent with the observed WSN/TN values, which confirms its suitability as an indicator of ripening in *Serpa* cheese. The technological landscape of *Serpa* cheese is characterised by significant heterogeneity between artisanal and semi-industrial processes. Roseiro et al. [147] noted that, while both production methods (PDO and non-PDO) generally align with national regulations, they differ significantly in maturation indices and moisture content. This variability was further explored by Araújo-Rodrigues et al. [142], who identified a high chemical diversity influenced by the specific dairy, month, and season of production. Despite this variance, the study pinpointed 13 specific chemical markers, including certain free amino acids (Tyr, Trp, Ile), volatile fatty acids (C3, C4, iC5), and the elastic modulus, as essential indicators of *Serpa*’s authenticity.

Alvarenga et al. [148] demonstrated that the specific extract of *C. cardunculus* and specific dairy practices significantly shape the nitrogen fractions and fatty acid profiles, dominated by palmitic, myristic, and oleic acids during the 30-day ripening period.

Finally, the relationship between chemical composition and consumer perception has been clarified through advanced sensory mapping. Macedo et al. [87] utilised solid-phase micro-extraction–gas chromatography–mass spectrometry (SPME-GC-MS) and discriminant analysis modelling to correlate volatile organic compounds with expert sensory scores. Their findings revealed that high-quality flavour profiles are associated with compounds like acetoin and diacetyl, whereas lower sensory scores are linked to an excess of 3-methylindole and capric acids.

Together, these studies provide a modern scientific framework to revise existing regulations and implement technological tools that safeguard the sensory heritage and safety of *Serpa* cheese, as reported by Araújo-Rodrigues et al. [149].

#### 2.2.7. Mestiço de Tolosa

*Mestiço de Tolosa* PGI is a mature cheese produced from raw ewe’s and goat’s milk blended in variable proportions (20:80, 40:60, or 60:40, respectively). Coagulation is achieved using *C. cardunculus*, followed by a ripening period of 3–4 weeks. During maturation, the cheese is immersed in a mixture of *pimento* powder and water, which imparts a characteristic orange-yellow colour to the rind. The cheese has a mildly strong, spicy, and pleasant flavour. These cheeses are small, with a diameter of 7–10 cm and a weight ranging from 150 to 400 g. *Mestiço de Tolosa* is the least studied Portuguese cheese, with available information limited to basic compositional parameters. Reported values indicate a moisture content of 55–65% in non-fat cheese and a fat content ranging from 45 to 65% on a dry basis.

## 3. New Technologies Applied to Iberian Small Ruminants’ Milk Cheese Production and Quality Control

Over the last ten years, several developments have occurred regarding the introduction of innovative approaches to safeguard the unique characteristics of Iberian small ruminants’ milk cheeses, guarantee their authenticity and develop tools to improve their quality and safety. However, further actions are needed to transfer research findings to the producers. As reviewed by Inácio et al. [150], recent advancements include the genetic screening of cardoon ecotypes, the inclusion of functional extracts in cheese and the application of high-pressure processing (HPP) to enhance safety. Collectively, these studies provide a comprehensive framework for understanding the biochemical and microbial dynamics of *Serra da Estrela* cheese, ensuring that this ancestral product remains competitive and safe in a modern global market.

Regarding cardoon quality control, Ordiales et al. [34] developed a PCR-based method to identify *C. cardunculus* genotypes used as coagulants in *Torta del Casar* manufacture. Analysis of 105 cardoon specimens in the *Extremadura* region revealed substantial genetic diversity, confirming that the plant rennet used in cheesemaking has distinct genetic profiles. This molecular approach was proposed as an effective tool for monitoring cardoon selection and improving control over texture variability in the final cheese. The best characterised expression system for recombinant cyprosin B was found to be *Saccharomyces cerevisiae*, although *Kluyveromyces lactis* has also been employed to secrete cardosin B, leading to the development of a preparation known as VRen [44]. VRen consists of a crude culture cardosin B-enriched extract that does not require purification and shows stability and proteolytic activity comparable to native cardosin B. Synthetic cardosin B, while less catalytically efficient, exhibited higher selective sequence, which may confer technological advantages in cheese production. More recently, recombinant forms of milk-clotting enzymes from cardoon flowers have been explored as alternatives to native extracts [151,152]. Nonetheless, the use of recombinant enzymes is still incompatible with PDO cheese regulations. Additionally, advanced analytical techniques are now being employed to monitor these processes with higher precision. From the use of free zone capillary electrophoresis to predict rennet impact on creaminess [10] to excitation–emission fluorescence matrices for the botanical and origin-based differentiation of cheeses [56], these tools provide a framework to enhance the consistency of cardoon in the production of these artisanal products.

The use of culture-independent (high-throughput sequencing, HTS) approaches to identify the main microorganisms responsible for the distinctive flavour characteristics of these cheeses opened an essential pathway to improve the standardisation of production and safety enhancement. The identification of the predominant bacteria in each type of cheese by metagenomic analysis allows for the production of “cheese-specific starter cultures”, which provides better control over the ripening process, with positive impacts on cheese quality, uniformity and safety. This method is probably the most significant tool to protect these products. The potential of OMICs technologies, namely metagenomics, in unravelling the complex microbial ecosystems of traditional Portuguese cheeses was discussed by Serrano et al. [98]. It is worth noting, however, that OMIC approach applications to Portuguese PDO cheeses are still scarce; current literature is limited to lipidomics and volatilomics (Reis Lima et al. [123]; Inácio et al., [153]), alongside a few metagenomic assessments (Rocha et al. [101]; Araújo-Rodrigues et al. [86]). Improved analytical tools associated with discriminant analysis demonstrated that FAA profiles can be effectively used to discriminate producers, geographical origin, and production periods [123]. These studies can play a decisive role in strengthening the authenticity of these cheeses as well as in differentiating PDO from non-PDO cheeses. Several tools have also been developed to evaluate the quality of these cheeses during ripening. Crespo et al. [154] utilised ultrasound parameters to identify manufacturing defects (e.g., improper pressing) in *Torta del Casar*. Martín-Tornero et al. [155] used fluorescence matrices (EEMs) to successfully differentiate between *La Serena* and *Torta del Casar* after 36 days of ripening based on tryptophan and retinol signals.

In addition, the use of autochthonous bioprotective cultures to address safety and consistency issues is crucial. It is worth mentioning that Martín et al. [156,157] demonstrated that adding *L. casei* 116 or *L. garvieae* 151 as bioprotective cultures significantly reduced *L. monocytogenes* in *La Serena* cheese without altering the cheese’s physical properties, potentially even enhancing the aroma profile. More recently, Araújo-Rodrigues et al. [142] successfully isolated and tested local LAB strains, finding that *L. plantarum* (PL1 and PL2) and *L. paracasei* (PC) could replicate the traditional lipolytic and proteolytic profiles of *Serpa* PDO cheese while improving standardisation. This shift towards “controlled tradition” can be complemented by innovations in coagulants.

Other ways to improve the safety of these cheeses may include the use of high-pressure processing (HPP). Garde et al. [158] evaluated the effect of HP treatment (300 or 400 MPa for 10 min) on *La Serena* cheeses at two ripening stages (day 2 and day 50). HP treatment applied on day 2 delayed casein degradation, resulting in a lower hydrophobic-to-hydrophilic peptide ratio and a higher total free amino acid content compared to untreated controls. By contrast, HP treatment on day 50 had little influence on proteolysis in cheeses ripened for 60 days. Regarding texture, cheeses treated at day 2 exhibited greater hardness, elasticity, and fracturability than either untreated controls or cheeses treated at day 50. Sensory analysis showed that cheeses subjected to 400 MPa at day 2 received the lowest flavour scores, whereas cheeses treated under other HP conditions did not differ significantly from the controls. Inácio et al. [153,159] reported that high-pressure processing (HPP) (400–600 MPa) slightly reduced LAB and total aerobic bacteria while eliminating Enterobacteriaceae, yeasts, moulds, and *L. innocua*. Physicochemical parameters were largely unaffected, and lipid oxidation was lower in HPP-treated cheeses, which indicates improved microbial safety and shelf life. Delgado Martinez et al. also evaluated the volatile compounds and sensory changes after high-pressure processing of mature *Torta del Casar* during refrigerated storage [160].

Brogueira [161] developed image-processing algorithms to classify *Évora* cheese based on eye formation, size, surface texture, and colour, enabling objective quality assessment. Dias et al. [162] evaluated the applicability of computer vision techniques as a novel method to evaluate gas-hole formation in *Nisa* cheese at different ripening stages (0, 15 and 35 days). Strong correlations were observed between image-derived parameters and physicochemical properties during ripening, particularly within the first 15 days, when major structural changes occurred. PCA confirmed a close association between image parameters and physicochemical evolution for up to 15 days of ripening. From 15 to 35 days, cheese evolution was mainly influenced by structural properties, such as elastic modulus and hardness. No significant effect of cardoon flower geographical origin on cheese structure was identified. Forty-eight samples were analysed over a 35-day ripening period using four different vegetable coagulants. Computer vision showed a high correlation with traditional chemical analysis during this phase. Interestingly, the geographical origin of the cardoon flower did not significantly influence the internal gas-hole patterns. The authors suggest that computer vision is a viable, objective method for monitoring cheese ripening and ensuring the structural consistency of *Nisa* PDO cheese. Dias et al. [163] investigated the influence of environmental conditions on *Évora* cheese ripening using experimental data and computational fluid dynamics (CFD) under steady-state conditions in ripening chambers. Air velocity and humidity were found to significantly affect physicochemical, microbiological, and sensory characteristics. Higher air velocity resulted in cheeses with lower moisture, higher MAB counts, a darker appearance, and more holes, while higher humidity was associated with lower scores for appearance, firmness, and odour intensity. CFD analyses enabled the identification of areas with uneven air distribution, which could impact maturation. Sensory evaluation indicated that locations promoting secondary microbial activity enhanced cheese paste neutralisation and increased aroma, flavour, and smell intensity while reducing hardness, highlighting the effect of factors beyond humidity on cheese quality. Locations with higher air velocity produced cheeses with lower moisture content and higher mesophilic bacteria counts. These cheeses typically appeared darker and had a higher frequency of internal holes. High-humidity environments negatively impacted sensory scores, particularly regarding appearance, firmness, and odour intensity. The CFD models identified specific areas within cheese stacks where air distribution was uneven. Mapping these “dead zones” allows producers to optimise room layouts to ensure a more uniform maturation process.

## 4. Challenges and Future Perspectives

Iberian PDO and PGI cheeses made with raw ewe’s and/or goat’s milk and coagulated with cardoon (*Cynara* spp.) are emblematic artisanal dairy products, with unique flavours, textures, and microbiomes shaped by indigenous LAB, yeasts, and fungi. Despite more than 40 years of research on their physicochemical and microbiological characteristics, the fundamental manufacturing practices have remained largely unchanged, with improvements primarily limited to better milk collection hygiene and sanitation in cheesemaking premises and processes. Consequently, although a wealth of data exists on these cheeses, significant process innovations or technological optimisations, although available, have not been applied due to the difficulty in transferring research results to cheese producers.

Recent reports highlight persistent challenges that affect cheeses’ quality and safety. Sensory defects caused by yeast and fungal activity are increasingly recognised. For example, *Yarrowia lipolytica* can induce visible browning reactions and subtle off-flavours or textural alterations. In addition, they can produce biogenic amines such as histamine and tyramine, which pose safety concerns. These issues underscore the need for more proactive management of the indigenous microbiota and spoilage organisms in traditional cheeses.

Additionally, the use of refrigerated milk has a significant impact on raw milk microbiota, and although it can reduce enterobacteria levels, the dominance of psychotropic bacteria in refrigerated milk poses several challenges as a result of their impact on proteolysis.

To ensure safety and quality in raw milk cheese production, several technological interventions have been explored. High-pressure processing (HPP) has proven effective in preventing over-ripening and excessive bitterness in mature cheeses, without requiring additional packaging steps.

To reconcile authenticity, safety, and economic sustainability, several strategies can be pursued:

***Standardisation and selection of elite cardoon ecotypes***

Variability in enzymatic activity among cardoon genotypes affects curd formation and subsequent proteolysis. The characterisation of the enzyme activity of different *Cynara* ecotypes for consistent coagulation and proteolysis is fundamental. It is important to maintain genetic diversity to preserve typicity. However, it is advisable to proceed to a systematic selection and propagation of high-performing ecotypes to reduce batch-to-batch variation while preserving characteristic flavours and textures.

***Optimisation of raw milk quality at the farm level***

Enhancing herd health and maintaining strict milking hygiene practices are essential to reduce the initial load of pathogens (e.g., *L. monocytogenes*, *S. aureus*) and spoilage microorganisms. On-farm rapid testing of somatic cell counts, total microbial load, and specific spoilage organisms can allow for milk selection before cheesemaking. Mild cooling protocols help control the growth of psychrotrophic bacteria while preserving the viability and activity of beneficial lactic acid bacteria. By optimising herd management, milking practices, and milk storage, contamination is minimised without compromising LAB populations, microbial succession during ripening is stabilised, and overall cheese consistency and quality are improved.

***Development of tailored starter and non-starter cultures***

The use of autochthonous LAB and yeast strains, either as starter cultures or adjunct bioprotective cultures, is a promising approach to enhance safety and quality. These cultures can inhibit undesirable microorganisms, reduce biogenic amine formation, and extend shelf life. In addition, they can maintain or improve cheeses’ organoleptic properties. Bioprotective cultures, derived from native microbiota, should ideally maintain antifungal and antibacterial activity throughout production and storage, not interfere with starter culture functionality, preserve the cheese’s sensory characteristics, be effective at low inoculum levels to reduce costs, and be easily propagated and resistant to freezing or freeze-drying. LAB are particularly promising due to their capacity to produce organic acids, bacteriocins, antifungal peptides, and other antimicrobial compounds, supporting both cheese safety and quality.

***Recognition of the social and economic impact of non-PDO cheeses***

Non-PDO cheeses represent a significant component of regional economies and local food culture, often produced by small-scale farms that rely on these products for income and employment. While they lack PDO certification, these cheeses frequently share the same raw materials, artisanal skills, and traditional practices as PDO cheeses. The strategic use of autochthonous starter and bioprotective cultures in non-PDO cheeses could markedly improve microbial safety, standardise quality, and enhance sensory consistency, increasing consumer confidence and marketability. By raising product quality without altering traditional methods, producers can access new markets, both domestically and internationally, while preserving the unique regional identity of their cheeses. Investing in non-PDO products also strengthens local economies, supporting small-ruminant dairy systems, reducing economic vulnerability, and diversifying income streams for rural communities. Far from competing with PDO cheeses, well-managed non-PDO products complement them, fostering overall resilience in artisanal cheesemaking regions and providing a buffer against market fluctuations or regulatory constraints.

***Risk-based regulatory approaches and PDO/PGI certification adjustments***

Current regulations often limit flexibility in artisanal cheesemaking. Incorporating risk-based frameworks that leverage modern microbiological insights, including metagenomics and predictive modelling, can help define safety thresholds while maintaining authenticity. Some certification criteria may require reformulation to integrate modern food-safety strategies and bioprotective interventions without undermining traditional production methods. Certification authorities need to reconsider rigid restrictions on starter cultures, as their judicious use can maintain authenticity while ensuring safety and consistency. Producers unwilling to adapt risk further declines in quality and competitiveness.

***Moving forward***

To preserve authenticity, ensure safety, and support rural economies, scientific knowledge must be translated into practical tools. Combining optimised milk hygiene, tailor-made starter and bioprotective cultures, and standardised coagulant use can stabilise ripening, preserve the sensory identity of PDO and PGI cheeses, and mitigate safety risks. Without adopting these strategies, both PDO and non-PDO producers risk stagnation, quality decline, and low competitiveness. Embracing innovation while respecting traditional cheesemaking practices is essential to safeguard the future of Iberian small-ruminant cheese production, enhancing marketability and reinforcing rural economies.

## Figures and Tables

**Table 1:** PDO and PGI cheeses of Spain and Portugal produced with ewe’s and goat’s milks or mixtures of such milks (Ministério de Agricultura, Pesca y Alimentación [11]; DGADR, [12]).

	Cheese Designation	Origin	Classification	Ripening Time (Days)	Texture	MNF ^(a)^ (%)	FDM ^(b)^ (%)
**(i)**	La Serena	Spain	PDO 1996	≥20	Soft to Semi-Hard	50	50
	Los Pedroches	Spain	PDO 1996	150–210	Semi-Hard/Hard	≤50	≥45
	Torta del Casar	Spain	PDO 1996	≥60	Soft	≤50	≤50
	Azeitão	Portugal	PDO 1996	≥16	Semi-Soft	63–69	45–60
	Évora	Portugal	PDO 1996	30–90	Semi-Hard/Hard	54–63; 49–56	45–60
	Castelo Branco	Portugal	PDO 1996	≥40 (>90 *Velho*)	Semi-Soft (Semi-Hard/Hard)	54–69 (49–56)	45–60
	Serra da Estrela	Portugal	PDO 1996	40–60 (>90 *Velho*)	Soft (Semi-Hard/Hard)	61–69 (49–56)	45–60 (60)
	Nisa	Portugal	PDO 1996	45–60	Semi-Hard	54–65	45–60
	Serpa	Portugal	PDO 1996	≥30	Semi-Soft	61–69	45–60
**(ii)**	Idiazabal	Spain	PDO 1996	≥60	Hard	≤45	≤45
	Manchego	Spain	PDO 1996	≥30–2 years	Semi-Hard/Hard	≤45	≤50
	Roncal	Spain	PDO 1996	≥120	Hard	≤40	≤45
	Zamorano	Spain	PDO 1993	60 (<1.5 kg); 120 (>1.5 kg)	Hard	≤45	≤45
	Terrincho	Portugal	PDO 1996	30 (>90 *Velho*)	Semi-Soft/Semi-Hard	35–60 (20–55)	25–50 (35–60)
**(iii)**	Los Ibores ^1^	Spain	PDO 2005	≥60	Semi-Hard	≤50	≥45
	Majorero	Spain	PDO 1996	8–20; 20–60; >60	Soft to Hard	50; 43; 37	52; 54; 55.5
	Murcia al Vino ^2^	Spain	PDO 2002	30 (<0.5 kg); ≥45 (>0.5 kg)	Semi-Hard/Hard	≤45	≤45
	Palmero	Spain	PDO 2002	1–270; >270	Soft/Semi-Hard/Hard	Variable	≤35
	Cabra Transmontano	Portugal	PDO 1994	≥60	Extra-Hard	25–35	45–60
**(iv)**	Amarelo Beira Baixa ^1^	Portugal	PDO 1996	40–50 (>90 *Velho*)	Semi-Hard (Semi-Hard/Hard)	54–69 (49–56)	45–60
	Picante Beira Baixa ^1^	Portugal	PDO 1996	120–180	Semi-Hard/Hard	49–63	35–60
	Rabaçal	Portugal	PDO 1996	>20	Semi-Hard	52–60	≤45
**(v)**	Flor de Guía ^3^	Spain	PDO 2010	15–60; >60	Semi-Hard/Hard	≥43	≤27.5
	Mestiço de Tolosa ^3^	Portugal	PGI 2000	21–28	Semi-Soft	55–65	45–60

(i) Raw sheep milk coagulated by cardoon; (ii) raw sheep milk coagulated by animal rennet; (iii) raw goat milk coagulated by animal rennet; (iv) raw mixed sheep/goat milk coagulated by animal rennet; (v) raw mixed sheep/goat or sheep–cow milk coagulated by cardoon. PDO: Protected Designation of Origin; PGI: Protected Geographical Indication. ^(a)^ Moisture in non-fat matter; ^(b)^ fat in dry matter (fat cheeses: >43–60%; extra-fat cheeses: >60%). Mestiço de Tolosa: mixed sheep/goat milk (20/80%; 40/60% or 60/40%); Flor de Guía: mixed sheep/cow milk (60/40%). ^1^ May also be produced with cardoon; ^2^ made with pasteurised milk; ^3^ may also be produced with calf rennet.

**Table 2:** Aspartic proteases isolated from *Cynara* spp. and their milk-clotting activities (MCAs) (adapted from Ben Amira et al. [45]).

Plant	Aqueous Extract (MCA)	Name	Class (Specific MCA)
*Cynara cardunculus*	0.131 ± 0.025 UAC/mL (1 h of maceration) 0.164 ± 0.024 UAC/mL (24 h of maceration)	Cardosins	Cardosin A (1160 RU/g)
			Cardosin B (7556 RU/g)
			Cardosin C
			Cardosin D
			Cardosin E
			Cardosin F
			Cardosin G
			Cardosin H
*Cynara scolymus*	60,000–70,000 RU/g	Cynarase	Cynarase A (30,000 RU/g)
			Cynarase B (100,000 RU/g)
			Cynarase C (30,000-40,000 RU/g)
*Cynara humilis*		Cardosin A-like	

RUs: rennet units or clotting activity units (1 RU = amount of enzyme needed to coagulate 10 mL low-heat processed skim milk at 30 °C in 100 s; FIL/IDF 157/1992).

**Table 3:** Specific cleavage sites by cardosins in ovine and caprine caseins (adapted from Ben Amira et al. [45]).

Caseins	Endopeptidase	αs1-Casein	β-Casein	κ-Casein
Ovine	Cardosin B	Leu_156_–Asp_157_	Leu_127_–Thr_128_	Phe_105_–Met_106_
		Trp_164_–Tyr_165_	Leu_165_–Ser_166_	
			Leu_190_–Tyr_191_	
Caprine	Cardosin A and B	Phe_153_–Tyr_154_	Leu_127_–Thr_128_	Lys_116_–Thr_117_
			Leu_190_–Tyr_191_	

**Table 4:** Compounds with potential impact on flavour identified in Iberic ewe’s milk cheeses produced with milk clotted by cardoon (after Ordiales et al. [66]; Delgado et al. [82]; Torres [83]; Ferreira et al. [84]; Partidário et al. [85]; Dahl et al. [69]; Araújo-Rodrigues et al. [86]; Macedo et al. [87]).

		La Serena	Torta del Casar	Castelo Branco	Évora	Serra da Estrela	Serpa			La Serena	Torta del Casar	Castelo Branco	Évora	Serra da Estrela	Serpa
	Compounds								Compounds						
**Acids**	Acetic ^1^		✓					**Esters** **^8,9^**	Benzoic acid methyl ester	✓					
	Butanoic (Butyric) ^2^	✓	✓	✓	✓	✓	✓		Acetic acid butyl ester		✓				
	Hexanoic (Caproic) ^2,3^	✓	✓	✓	✓	✓			Butanoic acid ethyl ester		✓				
	Octanoic (Caprilic) ^3^	✓	✓	✓	✓	✓			Ethyl hexanoate				✓		
	Pentanoic (Valeric) ^3,4^		✓	✓		✓			Ethyl decanoate				✓		
	Isovaleric (iC5:0) ^5^		✓		✓	✓	✓		2-Methylpentane			✓			
	Isobutyric					✓			3-Methylpentane			✓			
	Propanoic (Propionic) ^3^		✓	✓		✓			Ethyl acetate					✓	
	Decanoic (Capric)				✓	✓	✓		Ethyl-octanoate					✓	
	2,4 Hexadienoic				✓				Ethyl-decanoate					✓	
	Hex-3-enoic			✓				**Methyl ketones** **^8^**	2-Nonanone		✓			✓	
	2-Methylbutanoic			✓					2-Heptanone		✓				
	2-Methyl-propanoic ^4^			✓					Diacetyl ^7^					✓	✓
	Margaric (C17:0)		✓						2-Propanone		✓			✓	
	Linolenic (C18:3)								2-Pentanone		✓				
**Alcohols**	Ethanol		✓	✓		✓			2-Butanone		✓	✓		✓	
	Benzyl alcohol	✓							4-Methyl-2-pentanone					✓	
	3-Methyl butanol ^6^		✓	✓	✓	✓			2-Hexanone					✓	
	Phenyl-ethanol	✓							3-Hexanone					✓	
	2,3-Butanediol			✓	✓	✓			3-Heptanone		✓			✓	
	1-Butanol		✓						3-Hydroxy-2-butanone			✓		✓	
	2-Butanol		✓					**Aromatic compounds**	Phenol			✓			
	1-Pentanol		✓	✓		✓			Methylindole ^11^ (Skatole)						✓
	2-Hexanol		✓						Phenyl ethanol			✓			
	n-Propanol		✓						Benzoic acid			✓			
	2-Methylpropanol		✓						1-Phenylpropan-2-one			✓			
	1-Octanol			✓		✓		**Sulphur compounds** **^10^**	Methyldisulfanylmethane			✓			
	2-Octanol			✓					Meth-oxysulfonyloxymethane			✓			
	4-Octanol					✓		**Alicyclic compounds**	Methylcyclopentane			✓			
	Benzenemethanol		✓						1-Methyl-2-cyclohexene			✓			
	2,5-Dimetil-3-hexanol		✓					**Terpenoids** *	γ-Curcumene	✓					
	2-Pentanol				✓				α-Curcumene	✓					
	Phenylethanol ^6^				✓				α-Terpineol	✓					
	Methanol				✓				Verbenone	✓					
	Nonanol					✓									
**Aldehydes**	Acetoin					✓		**Benzenoids** **	Xylene isomers	✓					
	Octanal				✓		✓		Ethyl benzene	✓					
	Nonanal		✓		✓				Propyl benzene	✓					
	3-Methylbutanal ^8^		✓	✓											
	Acetaldehyde			✓											
	Safranal			✓		✓									

^1^ Vinegar; ^2^ pungent and rancid odours; ^3^ sheepy-like odour; ^4^ long-ripened cheese rancid, putrid and sweet notes; ^5^ rancid and buttery notes; ^6^ fresh, sweet odour; ^7^ buttery notes; ^8^ floral and fruity notes; ^9^ higher-quality ewe’s cheeses; ^10^ lower-quality ewe’s cheeses; ^11^ faecal odour. * Introduced mainly from animal feed; ** introduced mainly from *C. cardunculus*. ✓ = compound detected.

**Table 5:** Significative correlations found between compounds with potential impact on cheese flavour and main microbial groups found in *Serra da Estrela* cheese (Tavaria et al. [21]).

	Volatile Component	Microbial Group
Non-refrigerated milk cheeses	Acetaldehyde	Lactobacilli (+0.62)
	Acetic acid	Staphylococci (+0.55)
	Butyric acid	Enterococci (+0.53); Staphylococci (+0.64);
	Caproic acid	Enterobacteriaceae (+0.51); Yeasts (+0.53)
	Ethanol	Leuconostoc (+0.52); Yeasts (+0.57)
	Hexyl-acetate	Enterococci (−0.51); Yeasts (+0.50)
	Iso-valeric acid	Enterobacteriaceae (+0.55); Yeasts (+0.59)
Refrigerated milk cheeses	2,3-Butanediol	Enterobacteriaceae (−0.51); Lactococci (−0.59)
	2-Butanone	Yeasts (+0.55)
	2-Nonane	Enterobacteriaceae (−0.79); Yeasts (+0.55)
	Acetic acid	Yeasts (+0.56)
	Acetoin	Yeasts (+0.81)
	Butyric acid	Enterobacteriaceae (−0.53)
	Capric acid	Yeasts (+0.66)
	Caprylic acid	Yeasts (+0.59)
	Ethyl acetate	Leuconostoc (+0.64); Yeasts (+0.81)
	Ethyl decanoate	Enterobacteriaceae (−0.73); Enterococci (−0.55)
	Ethyl octanoate	Enterobacteriaceae (−0.54); Yeasts (+0.61)
	iso-Butyric acid	Staphylococci (−0.81)
	Nonanol	Enterobacteriaceae (+0.99)
	n-Propanol	Yeasts (+0.57)
	Valeric acid	Lactococci (−0.87); Lactobacilli (−0.54)

## Data Availability

No new data were created or analysed in this study. Data sharing is not applicable to this article.
